# Characterizing ecomorphological patterns in hyenids: a multivariate approach using postcanine dentition

**DOI:** 10.7717/peerj.6238

**Published:** 2019-01-11

**Authors:** Carlos Coca-Ortega, Juan Antonio Pérez-Claros

**Affiliations:** Departamento de Ecología y Geología, Universidad de Málaga, Málaga, Málaga, Spain

**Keywords:** Hyaenidae, Teeth, Evolution, Durophagy, Ecomorphology

## Abstract

We analyze the multivariate pattern of lower and upper cheek dentition for the family Hyaenidae along its evolutionary history. A total of 11,698 individual measurements of lengths and widths for the main postcanine teeth were collected for 54 extinct and three extant species of this family and analyzed by means of principal component analyses. Our results indicate that the functional aspects are better reflected by lower cheek dentition as a result of mosaic evolution. The multivariate structure captured by the three first principal components correspond to different adaptive strategies. The two first components characterize the main groups of ecomorphs, while hunting species separate from scavengers along the third axis. In the context of Hyaenidae, the post-canine cheek dentition of *Parahyaena brunnea* and *Hyaena hyaena* shows an extreme degree of specialization in scavenging.

## Introduction

Hyaenidae is a family of Neogene carnivores whose living representatives are the remnants of a once diverse group. In spite of their dog-like appearance, hyenas belong to the suborder Feliformia together with cats, mongooses and civets. Currently, only four species compose this family, the aardwolf (*Proteles cristatus*) and the spotted, striped and brown hyenas (*Crocuta crocuta, Hyaena hyaena* and *Parahyaena brunnea*, respectively). *Proteles cristatus* is a highly specialized termite eater with a greatly reduced dentition (Kruuk & Sands, 1972; Cooper & Skinner, 1979). The remaining three living members of this family, the striped hyaena, the brown hyaena and the spotted hyaena, exhibit cheek teeth adapted to cracking bones. Striped and brown hyenas are basically solitary scavengers (Rieger, 1981; Mills, 1982, 1990). In contrast, spotted hyenas live in large matrilineal social groups as cooperative hunters, playing an important ecological role as the top predators of the ecosystems they inhabit (Kruuk, 1972; Mills, 1990). Molecular studies by Koepfli et al. (2006) indicate that the aardwolf diverged ca. 10.6 Ma from its bone-cracking living relatives, while the divergence between *Crocuta* and *Hyaena* plus *Parahyaena* occurred ca. 8.6 Ma. According to Koepfli et al. (2006) *Hyaena* and *Parahyaena* diverged more recently (ca. 4.2 Ma). Hyenas are of significant paleontological interest not only because they are frequently found in fossil assemblages of the Old World since the Miocene but also because (particularly since the Lower Pleistocene) many of these assemblages have been accumulated by them (Turner, Antón & Werdelin, 2008; Palmqvist et al., 2011).

**Figure 1:**
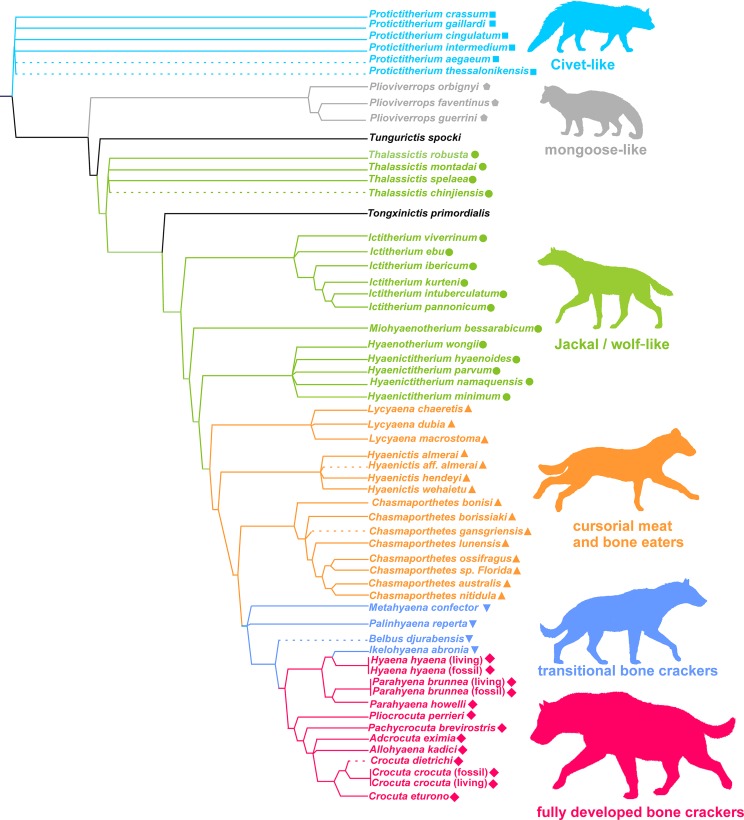
Phylogeny and adaptive types of Hyaenidae according to Turner, Antón & Werdelin (2008). Squares: civet-like insectivores/omnivores. Pentagons: mongoose-like insectivores/omnivores. Circles: jackal- and wolf-like meat and bone eaters. Triangles: cursorial meat and bone eaters. Inverted triangles: transitional bone crackers. Diamonds: fully developed bone crackers.

In agreement with Turner, Antón & Werdelin (2008), any investigations must operate within a clearly taxonomic framework. Although the taxonomic work on fossil hyenas has led to several important revisions (e.g., Howell & Petter, 1980; Kurtén & Werdelin, 1988; Werdelin, 1988a, 1988b; Semenov, 2008; Tseng, Li & Wang, 2013), the seminal study by Werdelin & Solounias (1991) represents the most comprehensive framework for this family. In clear contrast with its current status, Hyaenidae showed a high taxonomic diversity and ecological disparity in the past, with more than 70 described species. Hyenids are mainly known by their durophagous members, but durophagy is not exclusive to this family, nor were all hyenids bone crackers (Van Valkenburgh, 2007; Figueirido, Tseng & Martín-Serra, 2013). In spite of this family comprising more than 20 genera, practically all of them were assigned by Werdelin & Solounias (1996) to one of six ecomorphologies, which resemble living groups, namely: (1) civet-like insectivores/omnivores, (2) mongoose-like insectivores/omnivores, (3) jackal- and wolf-like meat and bone eaters, (4) cursorial meat and bone eaters, (5) transitional bone crackers and (6) fully developed bone crackers (Werdelin & Solounias, 1996; Turner, Antón & Werdelin, 2008). These ecomorphological groups or ecomorphs are based on qualitative traits and can be seen as groups of genera sharing a more or less similar functional guild (or adaptive zone *sensu* Van Valen, 1971) by morphological analogy with living groups. According to Turner, Antón & Werdelin (2008), these categories are successively evolved parts of the Hyenidae stem group (Fig. 1). Interestingly, although the cladistic characters used to construct the cladogram are different from those used to define ecomorphs (vg. position of infra-orbital foramen, position of anterior margin of orbit, suture between premaxillary and frontal on snout, etc.), those genera belonging to the same ecomorphological category cluster together in the phylogeny. In consequence, those two sets of characters must be related.

On the other hand, although some fossil hyenids have been the subject of detailed ecomorphological and biomechanical studies (e.g., Palmqvist et al., 2011; Tseng & Stynder, 2011; Tseng, Antón & Salesa, 2011), there has not been a quantitative study assessing if the cheek teeth reflect the ecomorphological spectrum of this family.

In this study, we depict a multivariate morphospace, in the same fashion to Pérez-Claros, Jiménez-Arenas & Palmqvist (2015), developed from metric variables of the postcanine dentition to specifically answer these questions: (1) Does the multivariate pattern of the cheek teeth capture the diversity of ecomorphs exhibited by this family in the past; and (2) are there multiple evolutionary trajectories to adapt to a given ecomorph? As we show below, the answer to both questions is affirmative.

## Materials and Methods

**Figure 2:**
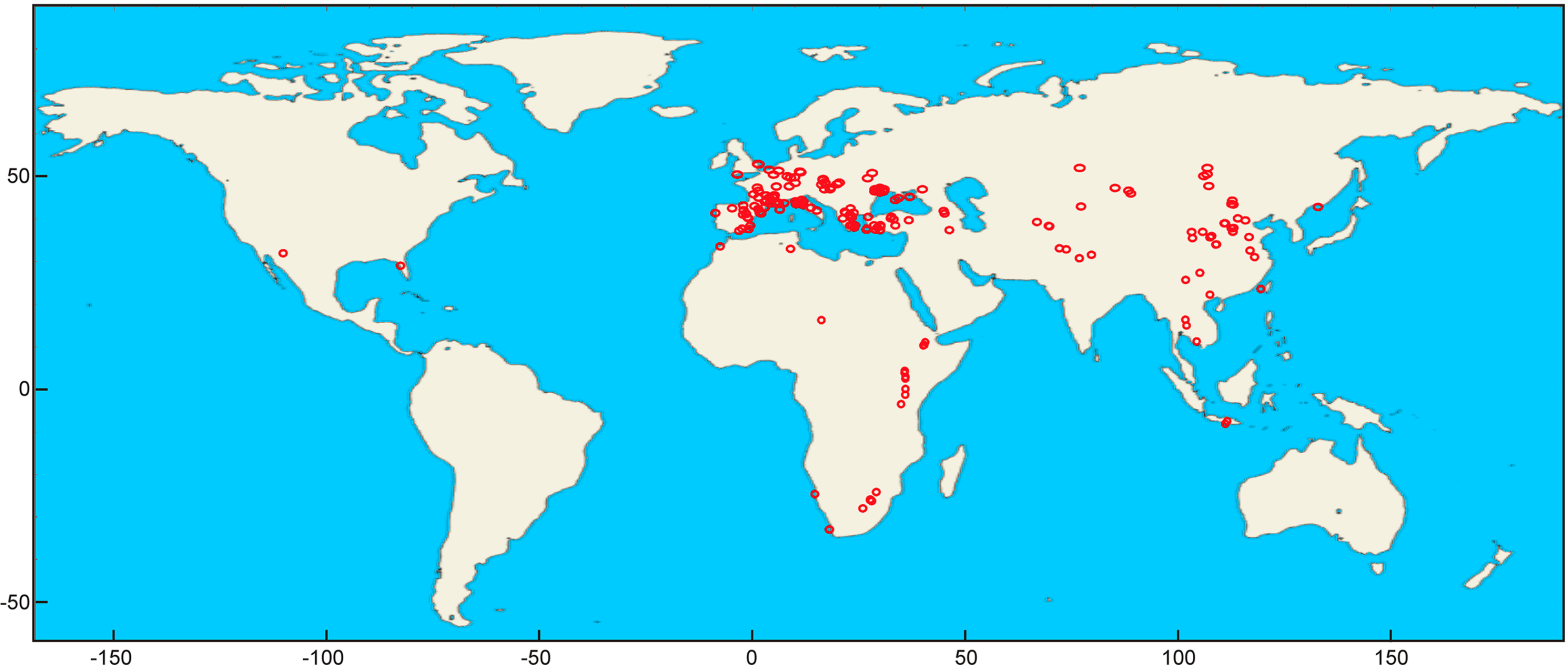
Fossil localities analyzed in the present study plotted using Mathematica (v. 10.4).

Our dataset consists of anteroposterior lengths (L) and buccolingual widths (W) for the lower (p3, p4 and m1) and the upper (P2, P3 and P4) cheek teeth of 60 species of hyenids covering the whole spectrum of adaptive types summarized in Turner, Antón & Werdelin (2008). Taxa have been considered valid according to Werdelin & Solounias (1991) and Turner, Antón & Werdelin (2008). Six new species of previously accepted genera and one of a new genus (*Werdelinus africanus*) described later have been assumed valid. Fossil representatives of living species have been analyzed separately, as they show some differences from their living counterparts, especially *Crocuta crocuta*, which encompasses many subspecies (e.g., *C. c. spelaea*, *C. c. praespelaea*, *C. c. ultra*, *C. c. angella*, *C. c. ultima* and other synonymized species of this genus). *Hyaena makapani* and *H. striata* have been assigned to *Hyaena hyaena* (fossil). The aardwolf, *Proteles cristatus*, is not analyzed here given its highly autapomorphic (and intraspecifically variable) dentition as a consequence of its ecological adaptation for termite eating. Data for fossils were mainly collected from 119 published sources (Data S1) comprising 415 fossil localities/cave members around the world (Fig. 2). Measurements for several fossils were taken from museum specimens (Data S2) using digital calipers to the nearest 0.1 mm. In some few cases, measurements were taken on figured specimens using tpsDig2 vers.2.26 (Rohlf, 2016).

Bivariate plots of width versus length for each dental element were used to detect outliers, which basically consisted of easily correctable typographical errors. However, there are some cases where the outliers were observations that are in some way different from, or inconsistent with, the rest of the data (Jolliffe, 2002, p. 233) but were not aliens to the sample to which they belong. Consequently, they are not wrong observations (atypical values *sensu* Reyment (1990), p. 128). Atypical values, although biologically perfectly acceptable, can adversely affect the performance of statistical procedures (Reyment, 1990, p. 128). That is the case of *Crocuta eturono*, whose teeth seen in isolation show no atypical proportions compared with other species of *Crocuta*. However, the relative lengths of its cheek teeth are quite different from any other *Crocuta* known (Werdelin & Lewis, 2008). This species has not been included to in the estimation of the principal components or discriminant analysis functions, although it has been projected on the morphospaces obtained using the rest of the observations.

A total of 11,698 individual measurements were collected (Data S3). The number of observations per variable ranges from 674 for WP2 to 1,221 for Lp4. The sample sizes for each variable and species are shown in Table 1. The number of observations per species and variable is very disparate, ranging from several hundreds to only one observation for the upper or the lower dentition for those poorly known taxa. In any case, the sample analyzed comprises practically all the described species of the family Hyenidae where the postcanine dentition is known. All the values for a given variable were averaged for each species (Data S4).

**Table 1:** Sample sizes for the species and the variables used in this study.

Id	Species	Ecomorph	Age	LP2	WP2	LP3	WP3	LP4	WP4	Lp3	Wp3	Lp4	Wp4	Lm1	Wm1
1	*Protictitherium aegaeum*	Civet-like	Tortonian-Messinian							1	1	1	1	1	1
2	*Protictitherium cingulatum*	Civet-like	Serravillian-Tortonian							1	1	1	1	1	1
3	*Protictitherium crassum*	Civet-like	Burdigalian-Tortonian	9	9	9	8	12	14	18	18	17	19	24	22
4	*Protictitherium gaillardi*	Civet-like	Burdigalian-Tortonian							3	2	5	4	5	5
5	*Protictitherium intermedium*	Civet-like	Langhian-Serravillian							2	2	4	4	11	11
6	*Protictitherium thessalonikensis*	Civet-like	Tortonian	4	4	5	5	4	4	1	1	1	1	1	1
7	*Tungurictis spocki*	–	Serravillian-Tortonian	3		3	3	3	3					1	1
8	*Plioviverrops faventinus*	Mongoose-like	Messinian-Zanclean	3	3	2	2	3	3	2	2	3	3	4	3
9	*Plioviverrops guerini*	Mongoose-like	Tortonian-Messinian							5	4	4	3	4	4
10	*Plioviverrops orbignyi*	Mongoose-like	Tortonian-Messinian	12	12	12	11	13	12	11	11	10	10	11	11
11	*Hyaenictitherium hyaenoides*	Jackal/wolf-like	Tortonian-Messinian	46	44	56	58	47	49	48	46	47	49	50	48
12	*Hyaenictitherium minimum*	Jackal/wolf-like	Messinian	3	3	4	4	2	1	7	7	9	9	10	8
13	*Hyaenictitherium namaquensis*	Jackal/wolf-like	Zanclean	2	2	3	3	4	4	3	3	3	3	3	3
14	*Hyaenictitherium parvum*	Jackal/wolf-like	Tortonian-Messinian	14	11	23	20	17	14	27	23	26	25	18	18
15	*Hyaenotherium wongii*	Jackal/wolf-like	Tortonian-Messinian	115	123	142	149	149	139	133	132	125	124	115	118
16	*Ictitherium ebu*	Jackal/wolf-like	Messinian	1	1	1	1	1	1						
17	*Ictitherium ibericum*	Jackal/wolf-like	Messinian-Zanclean	4	4	4	4	3	3	2	2	2	2	1	1
18	*Ictitherium intuberculatum*	Jackal/wolf-like	Tortonian	3	3	5	5	4	4	1	1	2	2	1	1
19	*Ictitherium kurteni*	Jackal/wolf-like	Tortonian-Messinian	1	1	1	1	1	1						
20	*Ictitherium pannonicum*	Jackal/wolf-like	Tortonian-Zanclean	1	1	2	2	3	3	11	7	9	7	8	7
21	*Ictitherium viverrinum*	Jackal/wolf-like	Tortonian-Zanclean	32	29	45	44	46	42	59	58	50	57	53	53
22	*Miohyaenotherium bessarabicum*	Jackal/wolf-like	Tortonian-Messinian	4	2	4	4	6	5	3	3	5	3	3	3
23	*Thalassictis chinjiensis*	Jackal/wolf-like	Serravillian-Tortonian							1	1	1	1	2	2
24	*Thalassictis montadai*	Jackal/wolf-like	Serravillian-Tortonian	2	2	2	2	2	2	5	4	4	3	5	5
25	*Thalassictis robusta*	Jackal/wolf-like	Tortonian	2	1	2	2	3	3	4	4	5	5	5	5
26	*Thalassictis spelaea*	Jackal/wolf-like	Tortonian	9	9	12	12	11	12	13	13	10	10	13	13
27	*Chasmaporthetes australis*	Cursorial bone-meat eater	Zanclean	3	3	4	4	3	3	6	6	7	7	6	6
28	*Chasmaporthetes bonisi*	Cursorial bone-meat eater	Tortonian-Zanclean	2	2	2	2	2	2	3	3	3	3	1	2
29	*Chasmaporthetes borissiaki*	Cursorial bone-meat eater	Zanclean	2	2	2	2	2	2	1	1	1	1	1	1
30	*Chasmaporthetes gansgriensis*	Cursorial bone-meat eater	Zanclean	2	2	1	1	1	1	2	2	2	2	2	1
31	*Chasmaporthetes lunensis*	Cursorial bone-meat eater	Zanclean-Gelasian	25	25	36	36	40	36	27	25	32	32	32	34
32	*Chasmaporthetes nitidula*	Cursorial bone-meat eater	Zanclean-Lower Pleistocene							9	11	6	7	2	2
33	*Chasmaporthetes ossifragus*	Cursorial bone-meat eater	Zanclean-Upper Pleistocene							5	5	7	5	5	3
34	*Chasmaporthetes sp. Florida*	Cursorial bone-meat eater	Gelasian	3	3	2	2	1	1	4	4	4	4	5	5
35	*Hyaenictis* aff. *almerai*	Cursorial bone-meat eater	Tortonian	2	2	2	2	2	2	2	2	2	2	2	2
36	*Hyaenictis almerai*	Cursorial bone-meat eater	Tortonian-Messinian							1	1	1	1	1	1
37	*Hyaenictis hendeyi*	Cursorial bone-meat eater	Zanclean							3	3	5	5	3	3
38	*Hyaenictis wehaietu*	Cursorial bone-meat eater	Zanclean	1	1	1	1	1	1	3	2	4	3	4	4
39	*Lycyaena chaeretis*	Cursorial bone-meat eater	Tortonian-Messinian	2	2	4	4	4	3	6	5	9	9	6	5
40	*Lycyaena dubia*	Cursorial bone-meat eater	Tortonian-Messinian	8	9	10	10	8	10	4	6	5	5	4	4
41	*Lycyaena macrostoma*	Cursorial bone-meat eater	Tortonian-Messinian							2	2	2	2	2	2
42	*Werdelinus africanus*	–	Messinian-Zanclean							4	3	3	4	2	2
43	*Belbus djurabensis*	Transitional bone-cracker	Messinian							3	3	4	4	3	2
44	*Ikelohyaena abronia*	Transitional bone-cracker	Zanclean	8	8	9	9	6	9	16	18	21	23	20	21
45	*Metahyaena confector*	Transitional bone-cracker	Tortonian							1	1	1	1	1	1
46	*Palinhyaena reperta*	Transitional bone-cracker	Tortonian-Messinian	9	10	10	11	10	11	8	10	11	12	7	8
47	*Tongxinictis primordialis*	–	Langhian-Serravallian	2	2	2	2	1	1						
48	*Adcrocuta eximia*	Fully developed bone cracker	Tortonian-Zanclean	81	74	96	93	91	82	106	101	106	98	88	90
49	*Allohyaena kadici*	Fully developed bone cracker	Tortonian	6	7	5	6	2	3	11	10	12	11	15	16
50	*Crocuta crocuta* (fossil)	Fully developed bone cracker	Gelasian-Recent	123	100	188	150	166	138	284	223	303	244	263	228
51	*Crocuta crocuta* (living)	Fully developed bone cracker	Recent	19	19	19	19	19	19	19	19	19	19	19	19
52	*Crocuta dietrichi*	Fully developed bone cracker	Zanclean-Gelasian	3	3	7	6	3	3	19	20	15	16	13	12
53	*Crocuta eturono*	Fully developed bone cracker	Piazencian	1	1	2	2	1	1	1	1	1	1	1	1
54	*Hyaena hyaena* (fossil)	Fully developed bone cracker	Gelasian-Recent	22	21	30	32	29	27	21	22	24	22	20	22
55	*Hyaena hyaena* (living)	Fully developed bone cracker	Recent	17	17	17	17	17	17	16	16	16	16	16	16
56	*Pachycrocuta brevirostris*	Fully developed bone cracker	Piacenzian-Upper Pleistocene	45	38	58	48	55	49	108	90	108	92	90	83
57	*Parahyaena brunnea* (fossil)	Fully developed bone cracker	Gelasian-Recent	5	5	5	4	6	6	11	11	8	7	9	7
58	*Parahyaena howelli*	Fully developed bone cracker	Zanclean	2	2	3	2	3	3	7	7	5	7	4	5
59	*Parahyena brunnea* (living)	Fully developed bone cracker	Recent	15	15	15	15	15	15	15	15	15	15	15	15
60	*Pliocrocuta perrieri*	Fully developed bone cracker	Zanclean-Upper Pleistocene	45	37	59	47	66	51	103	94	115	104	103	91
	Number of species per variable			46	45	46	46	46	46	56	56	56	56	57	57
	Number of observations per variable			723	674	926	867	888	815	1,192	1,088	1,221	1,130	1,115	1,059

**Note:**

Ecomorphs according to Turner, Antón & Werdelin (2008). Ages are according to standard chronostratigraphic units. LP2, LP3, and LP4: lengths of the second, third and fourth upper premolars. WP2, WP3, and WP4: widths of the second, third and fourth upper premolars. Lp3, Lp4, and Lm1: lengths of the third and fourth lower premolars and the first lower molar. Wp3, Wp4, and Wm1: widths of the third and fourth lower premolars and the first lower molar, respectively.

The species studied here were initially assigned to the ecomorphological group (adaptive types) of their respective genera according to Turner, Antón & Werdelin (2008). The basal hyenids *Tungurictis spocki* and *Tongxinictis primordialis* as well as *Werdelinus africanus* were not initially allocated to ecomorphs.

The principal component analyses were performed using the means for the lengths and widths of (i) only the upper dentition, (ii) only the lower dentition and (iii) the lower and upper dentition (to take into account the covariation between the two sets of variables). Given that some species have known values for only the upper or the lower dentition, the number of observations in each analysis is different (41, 44 and 55, respectively). Eigenvectors were computed from variance-covariance matrices using PAST v. 2.17 (Hammer, Harper & Ryan, 2001) since the variables analyzed were measured in the same units and using covariances gives more weight to those aspects with more variability.

Phylomorphospaces were generated to assess the phylogenetic signal in the principal components using the PDAP package (Midford, Garland & Maddison, 2011) in Mesquite (Maddison & Maddison, 2018). The reconstructed ancestral values were plotted, and the branches of the tree were connected (Sidlauskas, 2008; Klingenberg & Gidaszewski, 2010; Figueirido, Tseng & Martín-Serra, 2013). We use the tree topology published by Turner, Antón & Werdelin (2008), assuming branch lengths equal to one (vg. Grohé et al., 2016). In this framework, a strong phylogenetic signal leads to closely related species that tend to be near each other in the morphospace defined by the principal components. To test the presence of a phylogenetic signal in the data, we used the permutation approach presented by Klingenberg & Gidaszewski (2010), which simulates the null hypothesis that there is no phylogenetic signal by randomly interchanging each set of morphometric descriptors among the terminal nodes of the phylogeny (10,000 randomization runs per test).

Discriminant analyses were performed using SPSS v. 15.0.1 using the scores on the principal components as variables, given that, by definition, they are not correlated and, at the same time, the ratio of variables to the sample size is lower, which is more adequate since classification techniques require many more organisms than variables (Mitteroecker & Bookstein, 2011).

To facilitate the location of the species on the scatter plots, the name of each species in the text is followed by a number between brackets that corresponds to its numbering in Table 1.

## Results

The permutation test for a phylogenetic signal in the three datasets was statistically significant (*p* < 0.001), which indicates that there was a phylogenetic structure in the data.

The underlying multivariate pattern shown by the principal component analyses is readily interpretable and similar for the upper and lower postcanine dentitions (Table 2). For the three analyses, there is a first component explaining 96–97% of the variance, where all the variables show positive loadings, being clearly interpreted as a size axis. Obviously, size is the main source of variation, given that the sample ranges from animals similar to a mongoose to the giant hyaena, *Pachycrocuta brevirostris*, with an average estimated mass of ≈110 kg (Palmqvist et al., 2011). The second and third components scarcely explain 1.5% and 1% of the variance, respectively, although they are both very informative about the function of the hyenid cheek teeth. Similar results are obtained using the lower, the upper and the lower and upper postcanine dentition variables, which is evidenced by the high and very significant positive correlations between each principal axis and its corresponding homologue (Table 3). The weakest (although significant) correlation is obtained between the third components of the upper dentition and the lower dentition.

**Table 2:** Principal component loadings and variance explained for the three analyses.

	Variable	PC I	PC II	PC III
Upper and lower cheek teeth	LP2	0.232	−0.377	0.393
	WP2	0.185	0.193	0.038
	LP3	0.319	−0.109	0.374
	WP3	0.245	0.436	0.006
	LP4	0.527	−0.155	−0.164
	WP4	0.271	0.303	0.236
	Lp3	0.269	−0.054	0.187
	Wp3	0.224	0.515	−0.154
	Lp4	0.305	−0.267	0.162
	Wp4	0.199	0.257	−0.011
	Lm1	0.342	−0.304	−0.729
	Wm1	0.163	0.079	−0.063
	Eigenvalue	211.8	3.2	2.0
	% variance	96.3	1.5	0.9
Upper cheek teeth	LP2	0.300	−0.587	0.460
	WP2	0.240	0.229	0.089
	LP3	0.411	−0.196	0.417
	WP3	0.314	0.607	0.119
	LP4	0.681	−0.189	−0.702
	WP4	0.349	0.400	0.317
	Eigenvalue	121.7	1.7	0.9
	% variance	97.2	1.3	0.7
Lower cheek teeth	Lp3	0.437	−0.162	0.354
	Wp3	0.336	0.700	0.142
	Lp4	0.502	−0.523	0.393
	Wp4	0.307	0.373	0.164
	Lm1	0.537	−0.136	−0.819
	Wm1	0.248	0.231	−0.041
	Eigenvalue	105.3	1.6	1.2
	% variance	96.9	1.5	1.1

**Table 3:** Correlation between principal component scores for the three performed analyses.

		Upper and lower dentition			Upper dentition		
		PC I	PC II	PC III	PC I	PC II	PC III
Upper dentition	PC I	0.998**	0.004	0.032	–	–	–
	PC II	0.009	0.945**	−0.178	–	–	–
	PC III	0.015	0.105	0.806**	–	–	–
Lower dentition	PC I	0.996**	−0.009	−0.047	0.989**	0.014	−0.004
	PC II	0.209	0.907**	−0.151	0.214	0.799**	−0.022
	PC III	−0.087	0.351*	0.844**	−0.071	0.204	0.634**

**Notes:**

* Significant at 95%.

** Significant at 99%.

**Figure 3:**
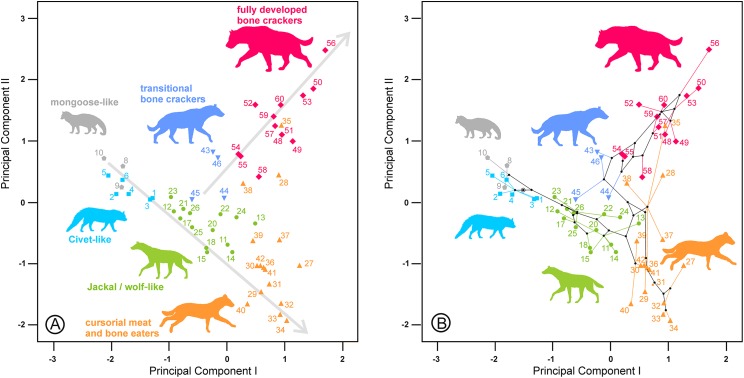
(A) Bivariate plot for the scores on the lower dentition two first principal components and (B) its corresponding phylomorphospace. The numbers correspond to the species in Table 1. Gray lines indicate allometric trends. Symbols as in Fig. 1.

**Figure 4:**
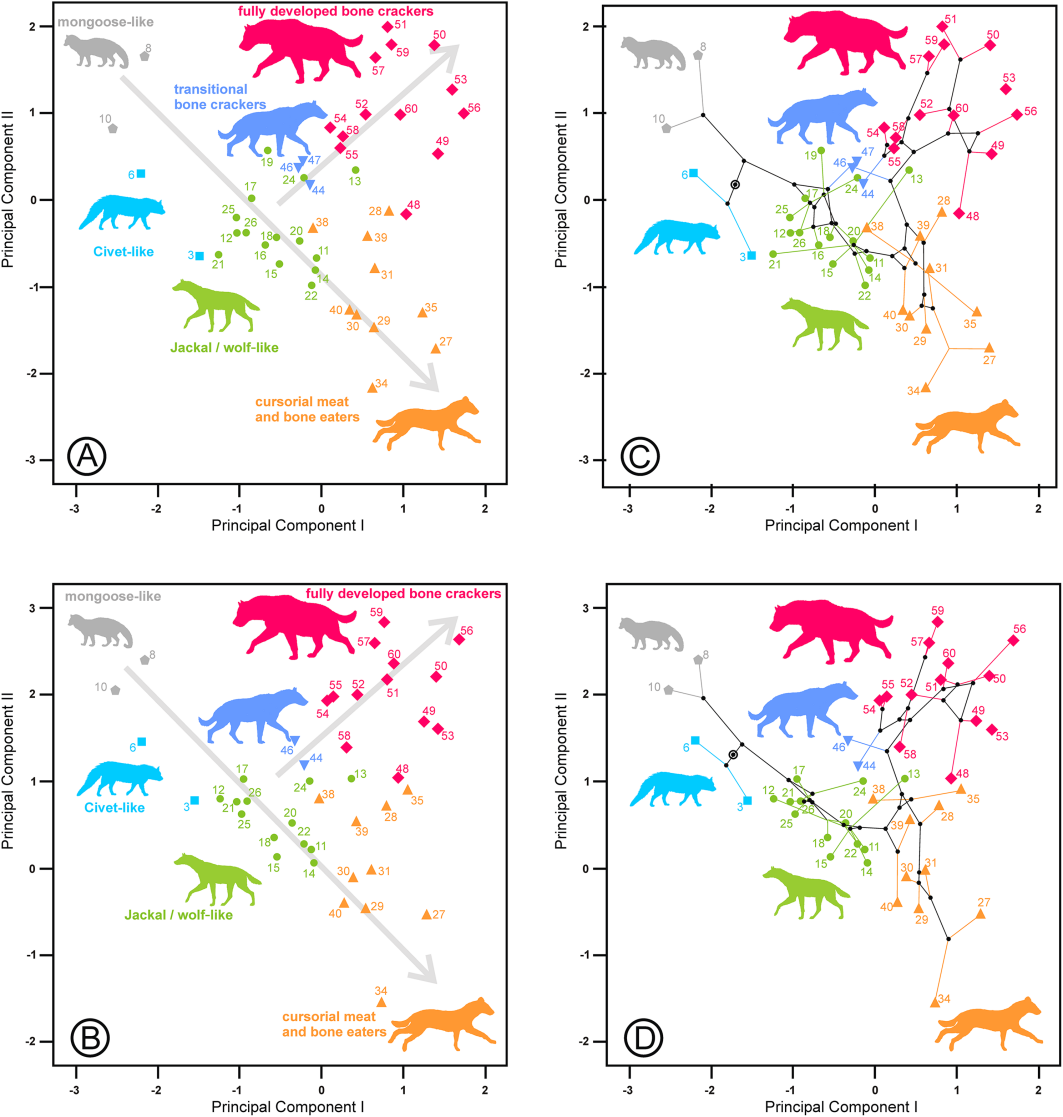
Bivariate plots of the scores on the two first principal components. (A) Upper dentition. (B) Upper and lower dentitions. (C) and (D) correspond to their phylomorphospaces, respectively. The numbers are as in Table 1. Gray lines indicate allometric trends. Symbols as in Fig. 1.

The second principal component in each analysis shows negative loadings for the lengths and positive loadings for the widths (Table 2). Along these shape axes, the dentitions are arranged from long and narrow shearing morphologies to more stoutly built teeth adapted to bone cracking. The width of the third upper and lower premolars have the highest component loadings on these components, which makes sense given that these teeth are the principal bone-cracking teeth (Werdelin & Solounias, 1991). LP2, WP4, Lp4 and Wp4 have relatively high correlations with the second components as well. However, the rest of the variables also play a more or less important role in defining the nature of this component. Plots of the species’ scores on components I and II for the lower, upper and upper and lower cheek teeth are in Figs. 3 and 4, respectively. From a visual inspection of these plots, the same general pattern may be observed, although it is clearer for the lower dentition, in part because there are more observations. A visual inspection of the phylomorphospaces (Figs. 3B, 4C and 4D) suggests that there is a clear phylogenetic signal, although there is some criss-crossing of branches. Species belonging to the same adaptive type or ecomorphological group (Werdelin & Solounias, 1996; Turner, Antón & Werdelin, 2008) share the same region of the morphospace defined by the two first components and, at the same time, they are arranged according to two well-defined morphological trends, which involve the two “post-thalassitine” major clades recognized by Werdelin & Solounias (1991). The first trend starts with the mongoose-like and civet-like hyenids (genera *Plioviverrops* and *Protictitherium*, respectively), which show the smallest cheek teeth, which are comparatively stouter than those of the more derived taxa belonging to the jackal- and wolf-like ecomorphs. This sequence ends with the cursorial meat-bone eater shearing and cutting morphologies, typical of the genus *Chasmaporthetes. Chasmaporthetes* sp. from Florida (#34) shows the most derived morphology for this morphological trend. The second trend starts with *Metahyaena confector* (#45), which is placed on the morphospace quite close to some species of the jackal- and wolf-like ecomorphs such as *Ictitherium viverrium* (#21) and *Thalassictis spelaea* (#26). Turner, Antón & Werdelin (2008) located *M. confector* in the transitional bone-cracker group only because its premolars show an incipient durophagous adaptation. This second morphological trend involves the rest of the transitional bone-cracker species and culminates with the fully developed bone crackers, whose more typical representative is *Pachycrocuta brevirostris* (#56). Fully developed bone crackers are basically aligned along this trend according to their sizes. There are, however, species mainly belonging to the cursorial meat and bone eater genera that plot for the lower dentition near the boundary between transitional and fully developed bone crackers (vg, *Chasmaporthetes bonisi* (#28) and *Hyaenictis wehaietu* (#38)) or even well inside the region of bone-cracking taxa such as *Hyaenictis* aff. *almerai* (#35), which are discussed below. Interestingly, *Hyaenictis* aff. *almerai* plots with cursorial meat and bone eater genera for the upper dentition (Fig. 4A). Likewise, *Hyaenictitherium namaquensis* (#13) (belonging to a wolf-like genus) is close to the cursorial meat and bone eaters for the two first lower dentition principal components, but it plots near fully developed bone crackers for the upper cheek teeth (Fig. 4A).

**Figure 5:**
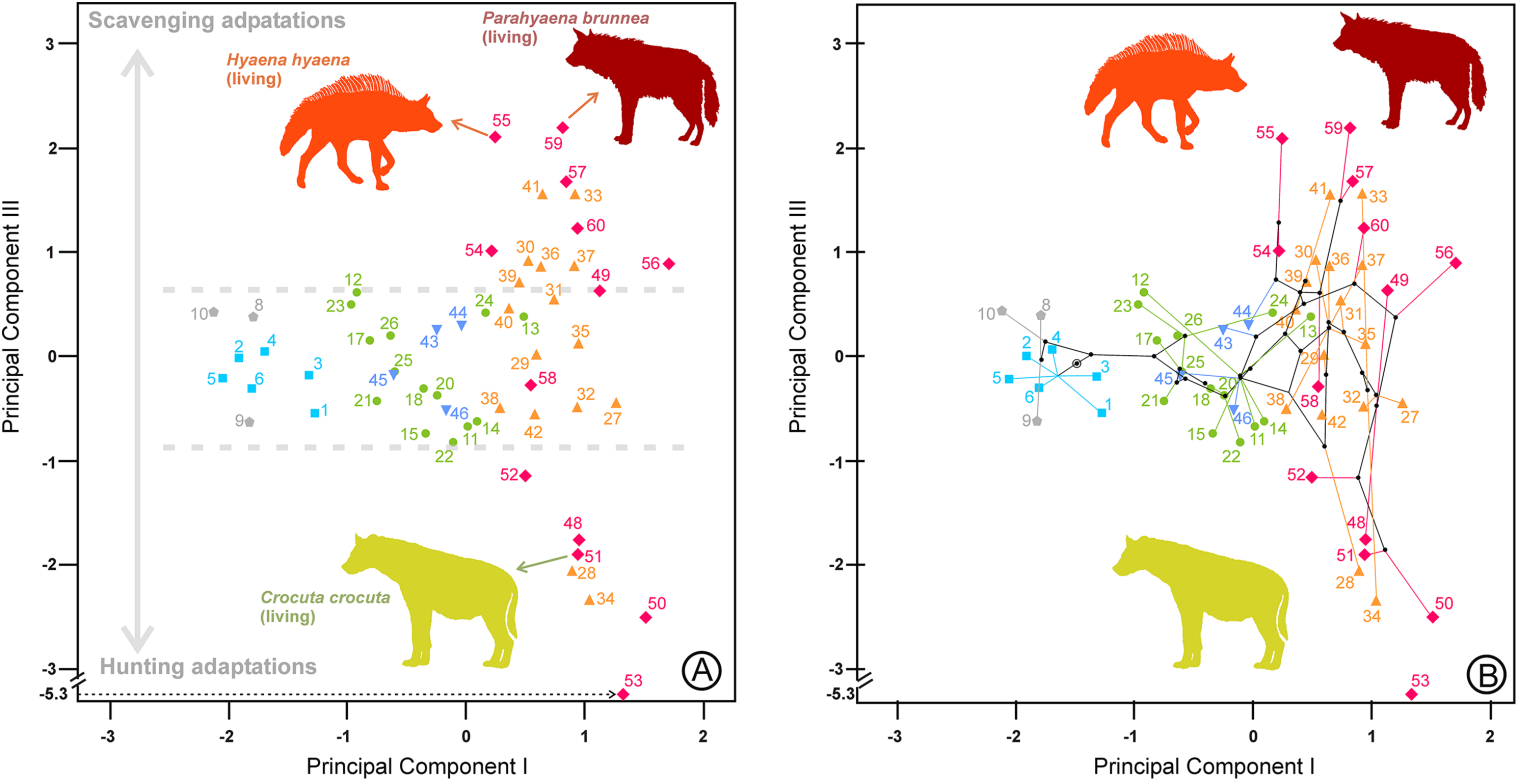
Bivariate plot of the scores on the (A) first and third principal components of the lower dentition and (B) its corresponding phylomorphospace. The numbers correspond to the species in Table 1. Symbols as in Fig. 1.

**Figure 6:**
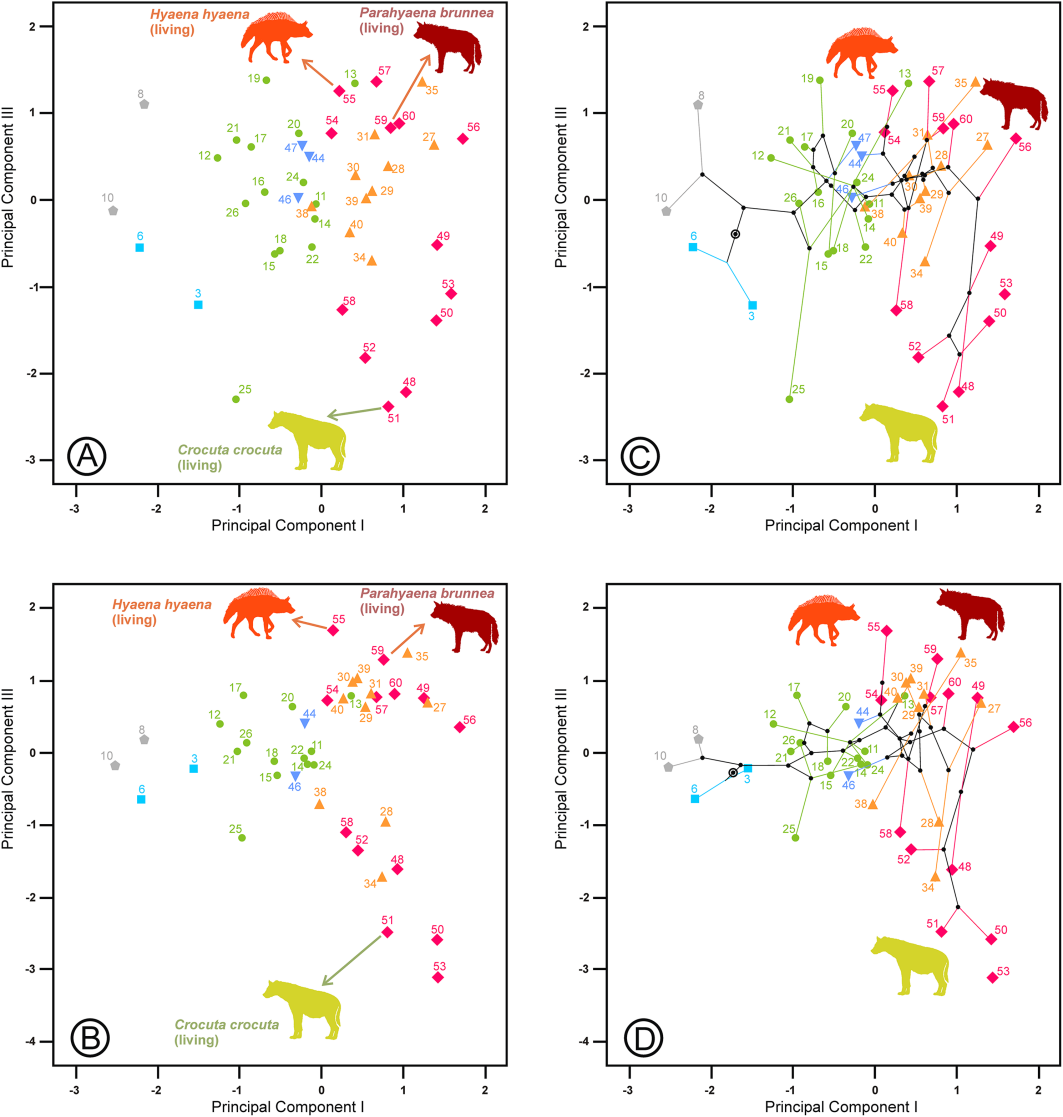
Bivariate plots of the scores on the first and third principal components. (A) Upper dentition. (B) Upper and lower dentitions. (C) and (D) correspond to their phylomorphospaces, respectively. The numbers are as in Table 1. Symbols as in Fig. 1.

The third component is characterized by its high negative correlation with the length of the lower carnassial tooth (Lm1) for the lower and upper and lower dentition analyses and with the length of its analogous tooth for the upper dentition analysis (LP4). However, at the same time, it is positively correlated with the lengths of P2 and P3 and their equivalents for the lower dentition, Lp3 and Lp4, which correspond to the length of the bone-cracking teeth in durophagous species (Table 2). Interestingly, the tooth widths are not clearly related to these third components. Figure 5 shows species scores on the first and third components. Some bone-cracking species as well as some cursorial meat and bone eaters take extreme, opposite values along the third component, while the rest of the ecomorphs show intermediate values. The interpretation of the third components can be performed with the help of the extant species, which are placed at the opposite ends of the morphospace (Figs. 5 and 6). First, the living species (and their fossil counterparts) with scavenger adaptations, namely, *Hyaena hyaena* (#55) and *Parahyaena brunnea* (#59) are located at the top, while *Crocuta crocuta* (#51), which is also an active predator (Kruuk, 1972; Mills, 1990), is at the bottom (jointly with its fossil representatives). Taking as a reference the most extreme values of the jackal- and wolf-like ecomorphs (Fig. 5), *Pliocrocuta perrieri* (#60) and *Pachycrocuta brevirostris* (#56) are located near the scavengers, while *Crocuta dietrichi* (#52) and *Adcrocuta eximia* (#48) plot on the hunting side. These facts, in addition to other arguments (as niche partitioning, see below), show that the third component seems somehow to reflect the adaptation for hunting or scavenging for fully developed bone cracking ecomorphs. *Crocuta eturono* (#53) projects as a typical fully developed bone cracker on the two first components, but it shows the most negative score on the third component, which might indicate a very pronounced hunting adaptation. Cursorial meat-bone eater species also project on both regions, perhaps as a consequence of the same type of adaptation, which is discussed in the next section. The third principal component of the upper dentition is something different from its counterpart for the lower cheek teeth, as evidenced by the correlation coefficient that is comparatively lower although still significant (*r* = 0.634, *p* < 0.001). The relative positions are more or less similar for fully developed bone crackers, but the other ecomorphs are much more scattered (compare Figs. 5, 6A and 6C). This is an interesting fact, indicating that this functional aspect is not equally reflected by the upper and lower cheek dentitions and so can be the result of mosaic evolution. The visual inspection of the phylomorphospaces (Figs. 5B, 6C and 6D) suggests that there is not a clear phylogenetic signal, as there is a high degree of criss-crossing of the branches.

A set of discriminant analyses using the scores on the principal components were performed to quantify the degree of proximity of each species to the center of its respective ecomorphological group as well as to assign an adaptive type to the non-allocated species. Table 4 shows the adaptive types assigned to each species according to the discriminant functions performed with the lower and upper dentitions, respectively. These results essentially confirm the graphical information shown by the principal components. There is a high degree of correspondence between the adaptive type according the genus and that assigned by the discriminant functions (85.2% and 83.7% of correct classifications using the scores for the lower and upper dentitions, respectively). Most of the disagreements are easily explainable, as they correspond with observations located at the boundary between ecomorphs, and slight changes in their respective positions can result in differences in assignment between neighbor groups. For example, in the case of civet-like and mongoose-like hyenids, the disagreement essentially resides in the position of *Plioviverrops guerini* (#9), which is located partially inside the cloud of civet-like hyenids. Something similar happens for those species that are just at the boundary between the jackal/wolf-like hyenids, cursorial bone-meat eaters and transitional bone crackers (vg., *Ictitherium kurteni* (#19), *Thalassictis montadai* (#24)*, T. robusta* (#25) or *M. confector* (#45)). However, there are other cases where there are noticeable differences in allocation according to the upper or lower dentition discriminant analyses such as *Hyaenictitherium namaquensis* (#13) and *Hyaenictis* aff. *almerai* (#35), which is discussed below.

**Table 4:** Results of the discriminant analyses.

Id	Species	Ecomorph according to genus	Ecomorph according to discriminant analysis (lower dentition)	Ecomorph according to discriminant analysis (upper dentition)
1	*Protictitherium aegaeum*	Civet-like	Civet-like	n/a
2	*Protictitherium cingulatum*	Civet-like	Civet-like	n/a
3	*Protictitherium crassum*	Civet-like	Civet-like	Civet-like
4	*Protictitherium gaillardi*	Civet-like	Civet-like	n/a
5	*Protictitherium intermedium*	Civet-like	**Mongoose-like**	n/a
6	*Protictitherium thessalonikensis*	Civet-like	**Mongoose-like**	Civet-like
7	*Tungurictis spocki*	–	Civet-like*	
8	*Plioviverrops faventinus*	Mongoose-like	Mongoose-like	Mongoose-like
9	*Plioviverrops guerini*	Mongoose-like	**Civet-like**	n/a
10	*Plioviverrops orbignyi*	Mongoose-like	Mongoose-like	Mongoose-like
11	*Hyaenictitherium hyaenoides*	Jackal/wolf-like	Jackal/wolf-like	Jackal/wolf-like
12	*Hyaenictitherium minimum*	Jackal/wolf-like	Jackal/wolf-like	Jackal/wolf-like
13	*Hyaenictitherium namaquensis*	Jackal/wolf-like	**Cursorial bone-meat eater**	**Transitional bone-cracker**
14	*Hyaenictitherium parvum*	Jackal/wolf-like	Jackal/wolf-like	Jackal/wolf-like
15	*Hyaenotherium wongii*	Jackal/wolf-like	Jackal/wolf-like	Jackal/wolf-like
16	*Ictitherium ebu*	Jackal/wolf-like	n/a	Jackal/wolf-like
17	*Ictitherium ibericum*	Jackal/wolf-like	Jackal/wolf-like	Jackal/wolf-like
18	*Ictitherium intuberculatum*	Jackal/wolf-like	Jackal/wolf-like	Jackal/wolf-like
19	*Ictitherium kurteni*	Jackal/wolf-like	n/a	**Transitional bone-cracker**
20	*Ictitherium pannonicum*	Jackal/wolf-like	Jackal/wolf-like	Jackal/wolf-like
21	*Ictitherium viverrinum*	Jackal/wolf-like	Jackal/wolf-like	Jackal/wolf-like
22	*Miohyaenotherium bessarabicum*	Jackal/wolf-like	Jackal/wolf-like	Jackal/wolf-like
23	*Thalassictis chinjiensis*	Jackal/wolf-like	Jackal/wolf-like	n/a
24	*Thalassictis montadai*	Jackal/wolf-like	Jackal/wolf-like	**Transitional bone-cracker**
25	*Thalassictis robusta*	Jackal/wolf-like	Jackal/wolf-like	**Civet-like**
26	*Thalassictis spelaea*	Jackal/wolf-like	Jackal/wolf-like	Jackal/wolf-like
27	*Chasmaporthetes australis*	Cursorial bone-meat eater	Cursorial bone-meat eater	Cursorial bone-meat eater
28	*Chasmaporthetes bonisi*	Cursorial bone-meat eater	**Fully developed bone cracker**	Cursorial bone-meat eater
29	*Chasmaporthetes borissiaki*	Cursorial bone-meat eater	Cursorial bone-meat eater	Cursorial bone-meat eater
30	*Chasmaporthetes gansgriensis*	Cursorial bone-meat eater	Cursorial bone-meat eater	Cursorial bone-meat eater
31	*Chasmaporthetes lunensis*	Cursorial bone-meat eater	Cursorial bone-meat eater	Cursorial bone-meat eater
32	*Chasmaporthetes nitidula*	Cursorial bone-meat eater	Cursorial bone-meat eater	n/a
33	*Chasmaporthetes ossifragus*	Cursorial bone-meat eater	Cursorial bone-meat eater	n/a
34	*Chasmaporthetes* sp. Florida	Cursorial bone-meat eater	Cursorial bone-meat eater	Cursorial bone-meat eater
35	*Hyaenictis* aff. *almerai*	Cursorial bone-meat eater	**Fully developed bone cracker**	Cursorial bone-meat eater
36	*Hyaenictis almerai*	Cursorial bone-meat eater	Cursorial bone-meat eater	n/a
37	*Hyaenictis hendeyi*	Cursorial bone-meat eater	Cursorial bone-meat eater	n/a
38	*Hyaenictis wehaietu*	Cursorial bone-meat eater	**Transitional bone-cracker**	**Jackal/wolf-like**
39	*Lycyaena chaeretis*	Cursorial bone-meat eater	Cursorial bone-meat eater	Cursorial bone-meat eater
40	*Lycyaena dubia*	Cursorial bone-meat eater	Cursorial bone-meat eater	Cursorial bone-meat eater
41	*Lycyaena macrostoma*	Cursorial bone-meat eater	Cursorial bone-meat eater	n/a
42	*Werdelinus africanus*	–	Cursorial bone-meat eater	n/a
43	*Belbus djurabensis*	Transitional bone-cracker	Transitional bone-cracker	n/a
44	*Ikelohyaena abronia*	Transitional bone-cracker	Transitional bone-cracker	Transitional bone-cracker
45	*Metahyaena confector*	Transitional bone-cracker	**Jackal/wolf-like**	n/a
46	*Palinhyaena reperta*	Transitional bone-cracker	Transitional bone-cracker	Transitional bone-cracker
47	*Tongxinictis primordialis*	–	n/a	Transitional bone-cracker
48	*Adcrocuta eximia*	Fully developed bone cracker	Fully developed bone cracker	Fully developed bone cracker
49	*Allohyaena kadici*	Fully developed bone cracker	Fully developed bone cracker	Fully developed bone cracker
50	*Crocuta crocuta* (fossil)	Fully developed bone cracker	Fully developed bone cracker	Fully developed bone cracker
51	*Crocuta crocuta* (living)	Fully developed bone cracker	Fully developed bone cracker	Fully developed bone cracker
52	*Crocuta dietrichi*	Fully developed bone cracker	Fully developed bone cracker	Fully developed bone cracker
53	*Crocuta eturono*	Fully developed bone cracker	Fully developed bone cracker	Fully developed bone cracker
54	*Hyaena hyaena* (fossil)	Fully developed bone cracker	Fully developed bone cracker	**Transitional bone-cracker**
55	*Hyaena hyaena* (living)	Fully developed bone cracker	Fully developed bone cracker	**Transitional bone-cracker**
56	*Pachycrocuta brevirostris*	Fully developed bone cracker	Fully developed bone cracker	Fully developed bone cracker
57	*Parahyaena brunnea* (fossil)	Fully developed bone cracker	Fully developed bone cracker	Fully developed bone cracker
58	*Parahyaena howelli*	Fully developed bone cracker	Fully developed bone cracker	Fully developed bone cracker
59	*Parahyena brunnea* (living))	Fully developed bone cracker	Fully developed bone cracker	Fully developed bone cracker
60	*Pliocrocuta perrieri*	Fully developed bone cracker	Fully developed bone cracker	Fully developed bone cracker

**Notes:**

Ecomorphs according to the genus by Turner, Antón & Werdelin (2008). Those entries written in bold are disagreements between the discriminant analysis assignment and the type corresponding to the genus.

* See the text for details.

Another interesting result is the adaptive assignment for the non-allocated genera. As shown by the upper dentition discriminant function, *Tongxinictis primordialis* (#47) is assigned to the transitional bone-cracker ecomorph (with a probability higher than 99.6%), and *Werdelinus africanus* (#42) is placed at the cursorial bone-meat eater group by the lower dentition discriminant function (probability of 97.2%). *Tungurictis spocki* (#7) is a very interesting taxon of uncertain status and functional adaptation (Werdelin & Solounias, 1991), although according to Wang (2004) this species is closely related to *Protictitherium gaillardi* (#4). Unfortunately, the width of its second upper premolar is unknown, and consequently it is not possible to obtain its scores on the upper dentition principal components. A discriminant analysis performed with the known variables for this species (LP2, LP3, WP3, LP4, WP4, Lm1 and Wm1) indicates a civet-like type for this taxon (with a probability of 99.7%), which is in accordance with Wang (2004).

**Figure 7:**
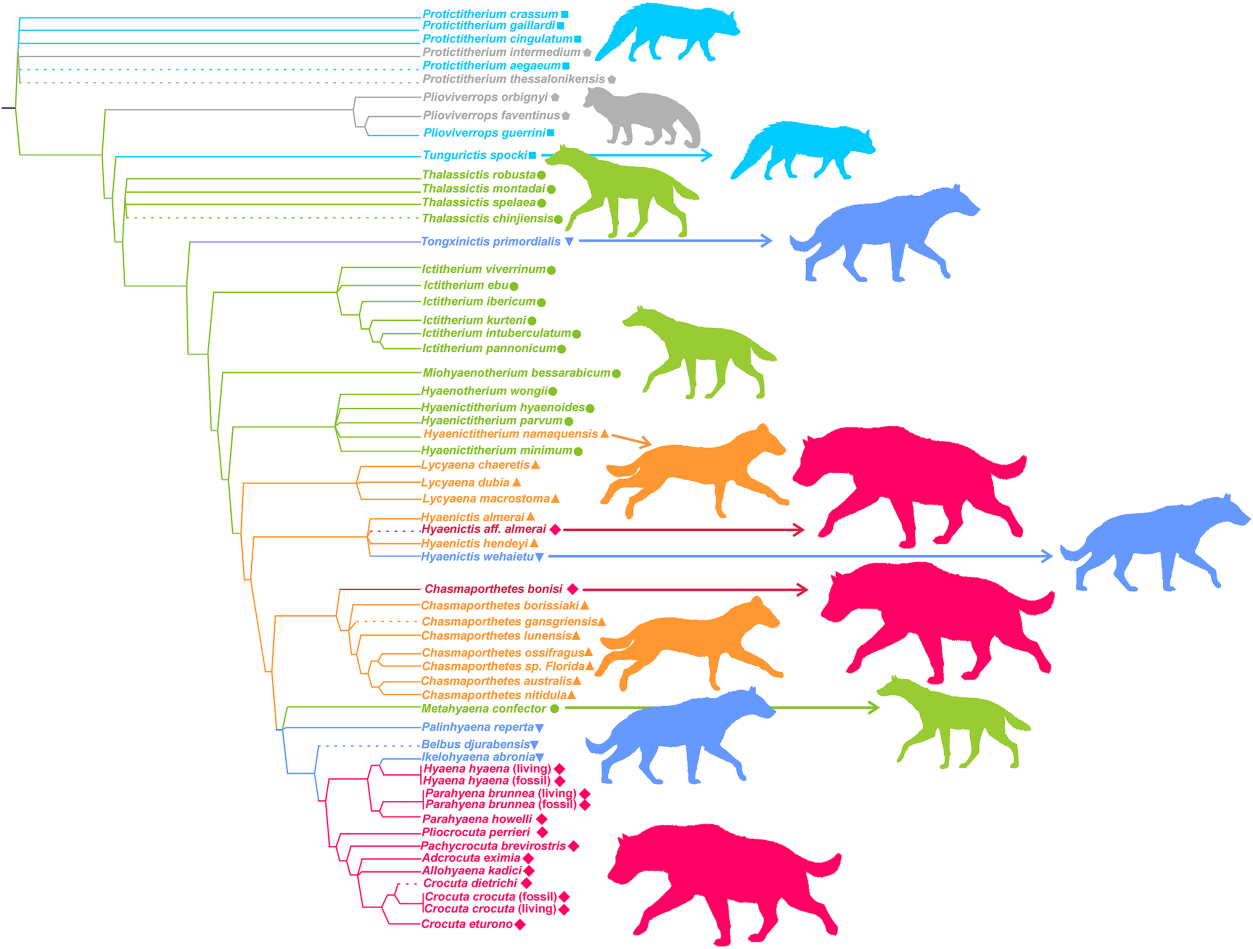
Phylogeny of Hyaenidae according to Turner, Antón & Werdelin (2008). Some new species have been tentatively allocated (dashed lines). The colors correspond to the adaptive types according to the lower dentition discriminant analysis. Colors and symbols as in Fig. 1.

Figure 7 shows the phylogeny of Hyaenidae according to Turner, Antón & Werdelin (2008), where some new species have been added and the adaptive types assigned by the lower dentition discriminant analysis are superimposed (obviously, with the exception of *Tongxinictis primordialis* (#47) and *Tungurictis spocki* (#7), whose ecomorphs are based on the upper dentition and upper and lower dentitions, respectively). The examination of the distribution of adaptive types on the cladogram shows a general agreement between the phylogenetic position and evolutionary type. However, there is not a linear sequence, as transitional bone crackers and fully developed bone crackers have evolved iteratively from different ancestors belonging to other functional categories. In the case of the transitional bone crackers, *Tongxinictis primordialis* (#47) and perhaps *Palinhyaena reperta* (#46) evolved from jackal- and wolf-like ancestors, while *Hyaenictis wehaietu* (#38) might come from cursorial bone-meat eaters (but see discussion). Fully developed bone crackers have appeared at least twice, in the case of *Hyaenictis* aff. *almerai* (#35) from cursorial bone-meat eaters and from transitional bone crackers for all the rest of the durophagous species (excluding *Chasmaporthetes bonisi* (#28), which can be synonymous with *Adcrocuta*, as discussed below).

## Discussion

The results obtained here indicate that the multivariate pattern defined by the two principal components of the postcanine dentition clearly captures the adaptive types for the hyenid genera devised by Werdelin & Solounias (1996) and summarized in Turner, Antón & Werdelin (2008). Turner, Antón & Werdelin (2008) suggested that this characterization probably requires some revision to the separation between civet-like and mongoose-like ecomorphs, which is also partially corroborated here, as only those two groups show a certain degree of overlap. However, according to the present study, this framework continues to provide a convenient overview of the postcanine dentition evolution of this family. Interestingly, the functional aspects are better reflected by the lower dentition variables than those for the upper dentition. This fact can be interpreted as a result of mosaic evolution, which has been corroborated by the evolution of the dental enamel microstructure in hyenids (Tseng, 2011). A differential degree of plasticity of the upper and lower dentitions provides an explanation for this fact. Carnivores show a lesser degree of morphological plasticity in the cranium than in the mandible. The cranium morphology is a trade-off between different functional demands (e.g., feeding, vision, olfactory sense and brain processing), while the mandible is only involved in food acquisition and processing (Figueirido et al., 2011). Perhaps this fact also translates into the cheek teeth, as the lower teeth seem to be more prone to reflect adaptations than the maxillary dentition. Consequently, the following discussion is mainly focused on the lower cheek dentition.

As shown in Fig. 3, ecomorphs align along two opposite morphological trends in the morphospace defined by the two first principal components. Given that the first component is a vector of size and the second one is a shape vector, those trends are allometric rules as well. An interesting point for both allometries is the positive relationship between size and specialization, as the most derived morphs (cursorial bone-meat eaters and fully developed bone crackers) also show the largest teeth. Werdelin & Solounias (1991) also noted a size trend between the position on the cladogram and the length of the upper carnassial (excluding *C. lunensis* and *C. borissiaki*), as this variable is strongly correlated with size and shows minimal variability according to Gingerich (1974). The ancestral state reconstructions also align along such allometric trend (Figs. 3B, 4C and 4D). This fact indicates that the evolution of the traits described by the two first components basically follows the routes connecting the main adaptive types. The allometries defined by the two principal components have an evolutionary origin, as they are the result of an interplay between developmental dynamics and selective factors. However, it is important to note here that the position along an allometric line does not necessarily indicate an ancestor-descendant sequence. In the same way, the absence of great leaps in the morphospace is not definitively indicative of a gradual evolution.

Figure 7 shows that there is a roughly correspondence between the adaptive type and position on the cladogram, as more derived taxa also have more specialized dentition. However, there are several exceptions, and thus there is not a fixed rule for the acquisition of a higher degree of specialization.

*Tongxinictis primordialis* (#47) is the first representative of the transitional bone cracker dentition, showing advanced dental characteristics, although in most other features it is quite primitive (Werdelin & Solounias, 1991). The age of this species (Langhian-Serravillian) is surprisingly old, and its ancestors have to belong to jackal/wolf-like ecomorphs. In any case, this does not involve a great morphological jump in the cheek teeth, as jackal/wolf-like and transitional bone-crackers are quite close in the morphospace. *Hyaenictis wehaietu* (#38) also shows a transitional bone cracker lower dentition and might independently come from cursorial bone-meat eaters. Nevertheless, it is probable that this species does not belong to this genus, as suggested by Vinuesa et al. (2017), as the lack of p1 and m2 and the presence of a well-developed m1 metaconid question its assignment to this genus. Consequently, it would be necessary to perform a deeper study of the taxonomic affinities of this species to clarify its evolution. The last group of phylogenetically related transitional bone cracker species (*Palinhyaena reperta* (#46), *Belbus djurabensis* (#43) and *Ikelohyaena abronia* (#44)) have an independent origin, which might be a jackal/wolf-like ecomorph as in the case of *Tongxinictis primordialis* (#47). The crucial point is that the most basal representative of this clade, *M. confector* (#45), shows a jackal/wolf like cheek dentition. As a matter of fact, Turner, Antón & Werdelin (2008) assigned this species to the transitional bone cracker type only because its premolars show an incipient form from the more bulbous shape that they have in *Belbus* and later transitional bone crackers. In other features, it is very similar to the jackal/wolf-like taxa. Consequently, if *M. confector* shared a common ancestor with *Chasmaporthetes* or other cursorial meat and bone eaters according to the cladogram, this would imply a reversion to a less specialized dentition and even a morphological leap (Fig. 3). With the independency of phylogenetic considerations, in our opinion, the jackal/wolf-like morphotypes are crucial for understanding the evolution of the cheek teeth of hyenids, as it seems that they have originated both cursorial meat and bone eaters and transitional bone cracker species. Its central position in the morphospace (both in size and in shape) allowed evolution in both directions.

Fully developed bone cracker dental morphs have also independently arisen twice or three times, which depends on the acceptance of *Chasmaporthetes bonisi* (#28) as a valid taxon. Werdelin & Solounias (1991) questioned this species and assigned it to *A. eximia* (#48). This taxon is also close to *A. eximia* in the morphospace defined by the upper cheek teeth (Fig. 4A). However, our results are not conclusive, as the discriminant analysis for the upper dentition assigns it to the cursorial morphotype.

Vinuesa et al. (2017) assigned *Hyaenictis* aff. *almerai* (#35) to the transitional bone cracker morphotype. However, our analyses show that its lower dentition is typical of the fully developed bone crackers (Fig. 3A). On the other hand, the upper cheek teeth of this species are those expected for a cursorial meat and bone eater (Fig. 4A), which clearly indicates a mosaic evolution. On the other hand, given that *Hyaenictis* aff. *almerai* and the rest of the phylogenetically related fully developed bone crackers show independent origins (Fig. 7), their bone cracker dentitions can be considered as evolutionary convergences. Our results are compatible with a gradual evolution for the majority of fully bone cracker species but not for *Hyaenictis* aff. *almerai* since close species of this genus of cursorial meat and bone eaters (*Hyaenictis* aff. *almerai* (#36) and *Hyaenictis hendeyi* (#37)) have very distant allocations in the morphospace for the lower dentition. This exemplifies that morphological continuity is not synonymous with gradual evolution.

The third component for the lower dentition is an unexpected axis of variation that allows us to discriminate between scavenging and hunting adaptations within the fully developed bone cracker ecomorph. The main element for its interpretation is obviously the opposite allocation of the recent and fossil specimens of the three extant durophagous species whose trophic ecology is known. Scavenging and hunting are simply part of a continuous spectrum (Turner, Antón & Werdelin, 2008), but the extant species of fully developed bone cracker hyenas show extreme differences in hunting behavior. In fact, spotted hyenas live in large matrilineal social groups whose members hunt cooperatively (Kruuk, 1972; Mills, 1990). The frequencies of prey killed and scavenged by *Crocuta crocuta* show differences between localities, although it can reach up to 95% as, for example, in Masai Mara (Cooper, Holekamp & Smale, 1999). Medium-sized (100–200 kg) species are the best represented in the carcasses consumed by this hyaena (Palmqvist et al., 2011). The brown and striped hyenas are primarily solitary scavengers, although they can also hunt opportunistically. Striped hyenas can predate on livestock even larger than themselves, such as donkeys or horses, although mainly feed on small animals (rodents, birds, reptiles, fish), carrion and vegetables such as seeds or leaves (Rieger, 1981; Leakey et al., 1999). The brown hyaena is predominantly a scavenger of all types of vertebrate remains and supplements its diet with wild fruits, insects, eggs of birds and the occasional small animal that it kills (Mills, 1982). Vertebrate prey killed by *Parahyaena brunnea* contributed only 4.2% of its food items (Mills, 1982). In the Central Kalahari Desert, the remains of kills left by other predators are the single most important food item in the brown hyena’s diet (Owens & Owens, 1978). These differences in foraging behavior seem to be reflected in the lengths of their carnassials and bone-cracking teeth, captured by the third principal components. Under this scenario, fully developed bone crackers could specialize in two opposite directions: scavenging or hunting. It is important to note here that this type of adaptation is independent of the adaptative ecomorphs devised by Werdelin & Solounias (1996). Fossil representatives of *Hyaena hyaena* (#54) and *Parahyaena brunnea* (#57) seem to be less specialized in scavenging than their living counterparts, as they occupy a more central region (Fig. 5). In the case of the genus *Parahyaena*, this also holds for the extinct species of this lineage *Parahyaena howelli* (#58). *Pachycrocuta brevirostris* (#56), with a strict scavenging behavior according to Palmqvist et al. (2011), is also in the same region of *Hyaena* and *Parahyaena*. Interestingly, this species does not show an extreme dental morphology. Maybe its large size can be considered an adaptation for scavenging itself, given that its enormous size translates to a greater ability for demolishing bones than in the case of the brown and striped hyenas. This can also be deduced from the high percentage of unidentifiable bone shafts and fragments in the maternity dens (Palmqvist et al., 2011). *Pliocrocuta perrieri* (#60) is also placed on the scavenging side of this morphospace, which is in accordance with its relative specialization to demolish bone (Antón et al., 2006; Turner, Antón & Werdelin, 2008). The co-occurrence of *Pliocrocuta perrieri* (#60) and *Chasmaporthetes lunensis* (#31) (conceived as a group-hunting predator of medium-sized ungulates by Antón et al., 2006) also suggests a scavenging behavior for *Pliocrocuta perrieri*, given that it is unlikely that species of a similar size and niche could coexist. All the species of *Crocuta* and *A. eximia* (#48) plot on the hunting region. The bone-smashing capabilities of *A. eximia* are obvious. However, Turner, Antón & Werdelin (2008) indicate that its upper carnassial is rather sectorial, with a reduced protocone, which may imply a rather high flesh content in its diet. Werdelin (1996) also indicates the possibility that this species was an active hunter. The recent representatives of *Crocuta crocuta* (#51) seem to have been slightly less active hunters than their fossil counterparts, as shown in Fig. 5. Nevertheless, this contention should be treated with a certain caution, as *Crocuta crocuta* (fossil) consists of a heterogeneous collection of subspecies that deserve a more detailed analysis. *Crocuta dietrichi* (#52) seems to be a less specialized member of this genus in hunting or scavenging, which is in accordance with the point of view of Werdelin & Lewis (2008). On the other hand, under this scenario, *Crocuta eturono* (#53) would be the most specialized hunter morphotype ever exhibited by a fully developed bone cracker. As indicated by Werdelin & Lewis (2008), its teeth, seen in isolation, show no distinctive features of morphology or proportions different from any other species of *Crocuta*. However, the relative lengths of its cheek teeth are different from those of any other *Crocuta* known. Interestingly, in several bivariate diagrams of length proportions shown by Werdelin & Lewis (2008), *C. eturono* deviates in a direction opposite to that of *Pachycrocuta*, which corresponds to its relative position along the third principal component. The sympatry between *C. eturono* and its coeval *C. dietrichi* during the Pliocene of eastern Africa could be explained by an absence of competition according to their respective positions along this scavenging/hunting principal component. Interestingly, phylomorphospaces show that most of the ancestral nodes have intermediate scores on the third principal components (Figs. 5B, 6C and 6D) and point out that the specializations in the two opposite directions of this axis are independently derived conditions (homoplasies).

Most of the cursorial meat and bone eaters are relatively clustered together in a central position on the third principal component for the lower dentition (Fig. 5). However, *Lycyaena macrostoma* (#41) and *Chasmaporthetes ossifragus* (#33) are grouped with *Hyaena* and *Parahyaena*, while *Chasmaporthetes* sp. from Florida (#34) is close to *Crocuta crocuta* (#50, #51). *Chasmaporthetes bonisi* (#28) is again close to *A. eximia* (#48). The functional meaning of the positions of these three species is not as direct. The case of *Lycyaena macrostoma* (#41) perhaps can be explained as a collateral effect of its broad lower carnassials (Werdelin & Solounias, 1991). However, *Chasmaporthetes ossifragus* (#33) and *Chasmaporthetes* sp. from Florida (#34) are closely related species occupying opposite allocations along this axis. Kurtén & Werdelin (1988) proposed a new species for the Florida specimens (previously assigned to *C. ossifragus* by Berta, 1981), on the basis of a long m1 relative to p4, which explains its low score on the third component (see Table 2). Tseng, Li & Wang (2013) indicate that, although the p4/m1 length ratio of the *Chasmaporthetes* sp. from Florida is extreme among North American specimens, intermediate values are found for *Chasmaporthetes* specimens from China and even postulate a separate dispersal to the New World from *C. ossifragus*. Interestingly, *C. ossifragus* and *C.* sp. from Florida are the only hyenids recorded in New World, and their extreme positions with respect to other species of *Chasmaporthetes* along the third components perhaps could be the result of a local evolution in North America as a consequence of the presence of borophagine canids adapted to durophagy. In this context, Tseng & Wang (2011) suggest the competitive exclusion between hyenids and canids as an ecological mechanism to explain the lack of intercontinental dispersal during the Miocene, in spite of that many other large, cursorial carnivorans achieved it. Perhaps the lengthening of m1 with respect to p4 in *Chasmaporthetes* sp. from Florida is the result of an anagenetic evolution increasing the shearing component of the dentition at the expense of the bone-cracking component via natural selection to avoid competition with bone cracking borophagines.

## Conclusions

The multivariate pattern captured by the principal components of the lengths and widths of the main elements of the postcanine dentition clearly correspond to different adaptive strategies in the family Hyaenidae. Although there is general agreement between the results obtained by using the lower or upper dentitions, the former seems to reflect better the functional aspects. The ecomorphs devised by Werdelin & Solounias (1996) and summarized in Turner, Antón & Werdelin (2008) are aligned in two distinctly continuous sequences along the morphospace defined by the two first principal components. Mongoose-like, civet-like, jackal- and wolf-like and, finally cursorial meat and bone eaters are part of the main branch. On the other hand, jackal- and wolf-like, transitional bone cracker and fully developed bone cracker hyenids are ordered along the second branch. Although there is general agreement between the phylogenetic position and adaptive type, transitional bone crackers and fully developed bone cracker hyenids have independently arisen on at least two occasions. On the other hand, the continuum seen in the morphology is not necessarily indicative of gradual evolution. With the independence of the general functional strategies defined by the mentioned ecomorphs, fully developed bone crackers are distributed along an orthogonal, and hence non-correlated, axis where hunting species separate from scavengers. In this scenario, the post-canine cheek dentitions of *Parahyaena brunnea* and *Hyaena hyaena* exhibit an extreme degree of specialization in scavenging.

## Supplemental Information

10.7717/peerj.6238/supp-1 Supplemental Information 1 Provenance of the data collected from the literature.

10.7717/peerj.6238/supp-2 Supplemental Information 2 Specimens measured from the Museum of Natural History, University of Florence.

10.7717/peerj.6238/supp-3 Supplemental Information 3 Raw data. Measurements are in mm. Those data corresponding to means are followed by sample sizes between brackets.

10.7717/peerj.6238/supp-4 Supplemental Information 4 Variable means for the species analyzed.

## Additional Information and Declarations

### Data Availability

The following information was supplied regarding data availability: The raw data are provided in Data S3.

## References

[ref-1] Antón M, Turner A, Salesa MJ, Morales J (2006). A complete skull of *Chasmaporthetes lunensis* (Carnivora, Hyaenidae) from the Spanish Pliocene site of La Puebla de Valverde (Teruel). Estudios Geológicos.

[ref-2] Berta A (1981). The Plio-Pleistocene hyaena *Chasmaporthetes ossifragus* from Florida. Journal of Vertebrate Paleontology.

[ref-3] Cooper SM, Holekamp KE, Smale L (1999). A seasonal feast: long-term analysis of feeding behaviour in the spotted hyaena (*Crocuta crocuta*). African Journal of Ecology.

[ref-4] Cooper RL, Skinner JD (1979). Importance of termites in the diet of the aardwolf Proteles cristatus in South Africa. South African Journal of Zoology.

[ref-5] Figueirido B, MacLeod N, Krieger J, De Renzi M, Pérez-Claros JA, Palmqvist P (2011). Constraint and adaptation in the evolution of carnivoran skull shape. Paleobiology.

[ref-6] Figueirido B, Tseng ZJ, Martín-Serra A (2013). Skull shape evolution in durophagous carnivorans. Evolution.

[ref-7] Gingerich PD (1974). Size variability of the teeth in living mammals and the diagnosis of closely related sympatric fossil species. Journal of Paleontology.

[ref-8] Grohé C, Tseng ZJ, Lebrun R, Boistel R, Flynn JJ (2016). Bony labyrinth shape variation in extant Carnivora: a case study of Musteloidea. Journal of Anatomy.

[ref-9] Hammer Ø, Harper DAT, Ryan PD (2001). PAST: Paleontological statistics software package for education and data analysis. Palaeontologia Electronica.

[ref-10] Howell FC, Petter G (1980). The *Pachycrocuta* and *Hyaena* lineages (Plio-Pleistocene and extant species of the *Hyaenidae*). Their relationships with Miocene ictitheres: *Palhyaena* and *Hyaenictitherium*. Geobios.

[ref-11] Jolliffe IT (2002). Principal component analysis.

[ref-12] Klingenberg CP, Gidaszewski NA (2010). Testing and quantifying phylogenetic signals and homoplasy in morphometric data. Systematic Biology.

[ref-13] Koepfli KP, Jenks SM, Eizirik E, Zahirpour T, Van Valkenburgh B, Wayne RK (2006). Molecular systematics of the Hyaenidae: relationships of a relictual lineage resolved by a molecular supermatrix. Molecular Phylogenetics and Evolution.

[ref-14] Kruuk H (1972). The spotted hyena.

[ref-15] Kruuk H, Sands WA (1972). The aardwolf (Proteles cristatm Sparrman) 1783 as predator of termites. African Journal of Ecology.

[ref-16] Kurtén B, Werdelin L (1988). A review of the genus *Chasmaporthetes* Hay, 1921 (Carnivora, Hyaenidae). Journal of Vertebrate Paleontology.

[ref-17] Leakey LN, Milledge SAH, Leakey SM, Edung J, Haynes P, Kiptoo DK, McGeorge A (1999). Diet of striped hyaena in northern Kenya. African Journal of Ecology.

[ref-18] Maddison WP, Maddison DR (2018). http://www.mesquiteproject.org.

[ref-19] Midford PE, Garland T, Maddison WP (2011). http://mesquiteproject.org/pdap_mesquite/index.html.

[ref-20] Mills MGL (1982). Hyaena brunnea. Mammalian Species.

[ref-21] Mills MGL (1990). Kalahari Hyenas: comparative behavior and ecology of two species.

[ref-22] Mitteroecker P, Bookstein F (2011). Linear discrimination, ordination, and the visualization of selection gradients in modern morphometrics. Evolutionary Biology.

[ref-23] Owens MJ, Owens DD (1978). Feeding ecology and its influence on social organization in brown hyenas (*Hyaena brunnea*, Thunberg) of the central Kalahari Desert. African Journal of Ecology.

[ref-24] Palmqvist P, Martínez-Navarro B, Pérez-Claros JA, Torregrosa V, Figueirido B, Jiménez-Arenas JM, Espigares MP, Ros-Montoya S, De Renzi M (2011). The giant hyena *Pachycrocuta brevirostris*: modelling the bone-cracking behavior of an extinct carnivore. Quaternary International.

[ref-25] Pérez-Claros JA, Jiménez-Arenas JM, Palmqvist P (2015). Neurocranium versus face: a morphometric approach with classical anthropometric variables for characterizing patterns of cranial integration in extant hominoids and extinct hominins. PLOS ONE.

[ref-26] Reyment RA (1990). Reification of classical multivariate statistical analysis in morphometry.

[ref-27] Rieger I (1981). Hyaena hyaena. Mammalian Species.

[ref-28] Rohlf FJ (2016). TpsDig version 2.26 (Tps_Digitize). http://life.bio.sunysb.edu/morph/soft-dataacq.html.

[ref-29] Semenov Y (2008). Taxonomical reappraisal of “ictitheres” (Mammalia, Carnivora) from the Late Miocene of Kenya. Comptes Rendus Palevol.

[ref-30] Sidlauskas B (2008). Continuous and arrested morphological diversification in sister clades of characiform fishes: a phylomorphospace approach. Evolution: International Journal of Organic Evolution.

[ref-31] Tseng ZJ (2011). Variation and implications of intra-dentition Hunter-Schreger band pattern in fossil hyaenids and canids (Carnivora, Mammalia). Journal of Vertebrate Paleontology.

[ref-32] Tseng ZJ, Antón M, Salesa MJ (2011). The evolution of the bone-cracking model in carnivorans: cranial functional morphology of the Plio-Pleistocene cursorial hyaenid *Chasmaporthetes lunensis* (Mammalia: Carnivora). Paleobiology.

[ref-33] Tseng ZJ, Li Q, Wang X (2013). A new cursorial hyena from Tibet, and analysis of biostratigraphy, paleozoogeography, and dental morphology of *Chasmaporthetes* (Mammalia, Carnivora). Journal of Vertebrate Paleontology.

[ref-34] Tseng ZJ, Stynder D (2011). Mosaic functionality in a transitional ecomorphology: skull biomechanics in stem Hyaeninae compared to modern South African carnivorans. Biological Journal of the Linnean Society.

[ref-35] Tseng ZJ, Wang X (2011). Do convergent ecomorphs evolve through convergent morphological pathways? Cranial shape evolution in fossil hyaenids and borophagine canids (Carnivora, Mammalia). Paleobiology.

[ref-36] Turner A, Antón M, Werdelin L (2008). Taxonomy and evolutionary patterns in the fossil Hyaenidae of Europe. Geobios.

[ref-37] Van Valen L (1971). Adaptive zones and the orders of mammals. Evolution.

[ref-38] Van Valkenburgh B (2007). Déjà vu: the evolution of feeding morphologies in the Carnivora. Integrative and Comparative Biology.

[ref-39] Vinuesa V, Madurell-Malapeira J, Werdelin L, Robles JM, Obradó P, Alba DM (2017). A new skull of Hyaenictis Gaudry, 1861 (Carnivora, Hyaenidae) shows incipient adaptations to durophagy. Journal of mammalian evolution.

[ref-40] Wang X (2004). New materials of Tungurictis (Hyaenidae, carnivora) from tunggur formation, Nei mongol. Vertebrata PalAsiatica.

[ref-41] Werdelin L (1988a). Studies of fossil hyaenids: the genera *Ictitherium* Roth & Wagner and *Sinictitherium* Kretzoi and a new species of *Ictitherium*. Zoological Journal of the Linnean Society.

[ref-42] Werdelin L (1988b). Studies of fossil hyaenas: the genera *Thalassictis* Gervais ex Nordmann, *Palhyaena Gervais*, *Hyaenictitherium* Kretzoi, *Lycyaena* Hensel and *Palinhyaena* Qiu, Huang & Guo. Zoological Journal of the Linnean Society.

[ref-43] Werdelin L (1996). Community-wide character displacement in Miocene hyaenas. Lethai.

[ref-44] Werdelin L, Lewis ME (2008). New species of *Crocuta* from the early Pliocene of Kenya, with an overview of early Pliocene hyenas of eastern Africa. Journal of Vertebrate Paleontology.

[ref-45] Werdelin L, Solounias N (1991). The Hyaenidae: taxonomy, systematics and evolution. Fossils and Strata.

[ref-46] Werdelin L, Solounias N, Bernor RL, Fahlbusch V, Rietschel S (1996). The evolutionary history of hyenas in Europe and western Asia during the Miocene. The Evolution of Western Eurasian Neogene Mammal Faunas.

