# Supplementary material for: Characterizing ecomorphological patterns in hyenids: a multivariate approach using postcanine dentition
Source: PeerJ. 2019 Jan 11;6:e6238. doi: 10.7717/peerj.6238 (PMC6330948; doi:10.7717/peerj.6238)
Supplement: Supplemental Information 1 [file peerj-07-6238-s001.docx]

| **Species** | **References** |
| --- | --- |
| *Adcrocuta eximia* | 22, 44, 52, 53, 58, 59, 82, 84, 85, 92, 105, 114 |
| *Allohyaena kadici* | 44 |
| *Belbus djurabensis* | 21 |
| *Chasmaporthetes bonisi* | 20, 52, 56, 111 |
| *Chasmaporthetes australis* | 22, 93, 107 |
| *Chasmaporthetes borissiaki* | 63 |
| *Chasmaporthetes gansgriensis* | 93 |
| *Chasmaporthetes lunensis* | 4, 5, 16, 25, 34, 50, 51, 63, 81, 88, 92, 93, 99 |
| *Chasmaporthetes nitidula* | 30, 37, 63, 74, 93, 97, 98 |
| *Chasmaporthetes ossifragus* | 63, 93 |
| *Chasmaporthetes sp. Florida* | 63, 93 |
| *Crocuta crocuta (fossil)* | 3, 7, 8, 10, 11, 15, 28, 29, 32, 39, 61, 62, 64, 80, 89, 91, 92 |
| *Crocuta dietrichi* | 36, 37, 71, 104 |
| *Crocuta eturono* | 106 |
| *Hyaena hyaena (fossil)* | 29, 43, 61, 80 |
| *Hyaenictis aff. almerai* | 100 |
| *Hyaenictis almerai* | 9 |
| *Hyaenictis hendeyi* | 42, 107 |
| *Hyaenictis wehaietu* | 38 |
| *Hyaenictitherium hyaenoides* | 23, 83, 94, 108, 117, 118 |
| *Hyaenictitherium minimum* | 21 |
| *Hyaenictitherium namaquensis* | 78, 107 |
| *Hyaenictitherium parvum* | 83, 113 |
| *Hyaenotherium wongii* | 24, 35, 52, 75, 83, 101, 108, 114, 117 |
| *Ictitherium ebu* | 113 |
| *Ictitherium ibericum* | 83 |
| *Ictitherium intuberculatum* | 75, 101 |
| *Ictitherium kurteni* | 109 |
| *Ictitherium pannonicum* | 2, 82, 83 |
| *Ictitherium viverrinum* | 24, 27, 35, 52, 66, 83, 109, 114, 115 |
| *Ikelohyaena abronia* | 37, 43, 78, 104, 107, 113 |
| *Lycyaena chaeretis* | 1, 2, 18, 35, 40, 75, 84 |
| *Lycyaena dubia* | 75, 108, 117 |
| *Lycyaena macrostoma* | 115 |
| *Metahyaena confector* | 101 |
| *Miohyaenotherium bessarabicum* | 83 |
| *Pachycrocuta brevirostris* | 7, 13, 14, 28, 43, 49, 50, 64, 65, 67, 72, 73, 77, 80, 86, 87, 95, 110, 119 |
| *Palinhyaena reperta* | 108 |
| *Parahyaena brunnea (fossil)* | 6, 29, 41, 60, 61, 96, 98 |
| *Parahyaena howelli* | 104, 112 |
| *Pliocrocuta perrieri* | 2, 7, 12, 16, 26, 37, 43, 48, 49, 62, 64, 67, 69, 87, 99, 116 |
| *Plioviverrops faventinus* | 2, 19 |
| *Plioviverrops gervaisi* | 19 |
| *Plioviverrops guerrini* | 2, 19, 52, 70 |
| *Plioviverrops orbignyi* | 19, 52, 53, 58 |
| *Protictitherium aegaeum* | 47 |
| *Protictitherium cingulatum* | 68 |
| *Protictitherium crassum* | 19, 27, 31, 52, 54, 55, 68, 76, 83, 101, 115 |
| *Protictitherium gaillardi* | 19, 31, 33, 54 |
| *Protictitherium intermedium* | 46, 52, 68, 102 |
| *Protictitherium thessalonikensis* | 19, 57 |
| *Thalassictis chinjiensis* | 102 |
| *Thalassictis montadai* | 17, 101 |
| *Thalassictis robusta* | 83 |
| *Thalassictis spelaea* | 24, 83 |
| *Tungurictis spocki* | 45, 103 |
| *Tongxinictis primordialis* | 79 |
| *Werdelinus africanus* | 21 |
| *Crocuta crocuta (living)* | 90 |
| *Hyena hyena (living)* | 90 |
| *Parahyena brunnea (living)* | 90 |

**References:**

1. Adrover R, Alcalá L, Mein P, Moissenet J, Orrios J. 1986 Mamíferos del Turoliense medio en la Rambla de Valdecebro (Teruel). *Estudios geológicos* 42:495-510.

2. Alcalá L. 1994. *Macromamíferos neógenos de la fosa de Alfambra-Teruel*. Fac. de Cienc. Biol. Thesis, Universidad Complutense de Madrid.

3. Altuna J, Mariezkurrena K. 2000. Macromamíferos del yacimiento de Labeko Koba (Arrasate, País Vasco). *Munibe Antropologia-Arkeologia* 52:107-181.

4. Antón M, Turner A, Salesa MJ, Morales J. 2006. A complete skull of Chasmaporthetes lunensis (Carnivora, Hyaenidae) from the Spanish Pliocene site of La Puebla de Valverde (Teruel). *Estudios Geológicos* 62:375-388. DOI: 10.3989/egeol.0662132

5. Argant A. 2004. Les Carnivores du gisement Pliocène final de Saint-Vallier (Drôme, France). *Geobios* 37:133-182. DOI: 10.1016/S0016-6995(04)80013-5

6. Arribas A, Garrido G. 2008. Hiénidos [Pachycrocuta brevirostris (Aymard, 1846) y Hyaena brunnea Thunberg, 1820] del yacimiento de Fonelas P-1 (cuenca de Guadix, Granada) Hyaenids [Pachycrocuta brevirostris (Aymard, 1846) and Hyaena brunnea Thunberg, 1820] from the Fonelas P-1 site. In: *Vertebrados del Plioceno Superior terminal en el suroeste de Europa: Fonelas P-1 y el Proyecto Fonelas*. Cuadernos del Museo Geominero 10:201-230.

7. Baryshnikov GF, Tsoukala E. 2010. New analysis of the Pleistocene carnivores from Petralona Cave (Macedonia, Greece) based on the Collection of the Thessaloniki Aristotle University. *Geobios* 43:389-402. DOI: 10.1016/j.geobios.2010.01.003

8. Baryshnikov GF. 2014. Late Pleistocene hyena Crocuta ultima ussurica (Mammalia: Carnivora: Hyaenidae) from the Paleolithic site in Geographical Society Cave in the Russian far east. *Proceedings of the Zoological Institute RAS* 318:197-225.

9. Bataller JR. 1921. Mamífers fóssils de Catalunya: nota paleontológica. *Butlletí de la Institució Catalana d'Història Natural* 1921:80-86.

10. Beden M, Guérin C. 1973. *Le gisement de vertébrés du Phnom Loang (province de Kampot, Cambodge): Faune pléistocène moyen terminal (Loangien)*. IRD Editions

11. Beke D. 2010. The fossil cave hyena of Goyet, Walsin and Hastière (Belgium): osteometry and taphonomy. D. Biology. Thesis, Universiteit Gent.

12. Bernsen JJ. 1932. Eine Revision der fossilen Säugetierfauna aus den Tonen von Tegelen. *Natuurhistorisch Maandblad* 21:153-158.

13. Brongersma LD. 1937. On fossil remains of a hyaenid from Java. *Zoologische Mededelingen, Rijksmuseum van Natuurlijke Historie te Leiden* 20:186-202.

14. Brugués RJ, De Villalta-Comella JF. 1984. El yacimiento de vertebrados del Pleistoceno inferior de Crespià (Girona, NE de la Península Ibérica). *Acta geológica hispánica* 19:129-138.

15. Cardoso JL. 1993. La hyène des" Oubliettes" de Gargas, Crocuta crocuta spelaea (Mammalia, Carnivora). *Bulletin du Muséum national d'histoire naturelle* 15:79-104.

16. Crusafont M, Aguirre E. 1971. Euryboas lunensis et Hyaena donnezani associées, en Espagne dans le gisement d’age Pliocène terminal de Layna (Soria). *Comptes Rendus de l'Académie des sciences, Paris, sèrie D* 273:2476-2478.

17. Crusafont M, Colpe M. 1973 Nuevos hallazgos de Progenetta montadai en el Mioceno de Cataluña. *Boletín Geológico y Minero* 84:105-113.

18. Crusafont M, Villalta JF. 1945. Hallazgo de la Lycyaena chaeretis GAUDRY, en el Mioceno de Saldaña. *Estudios Geológicos* 2:113-120.

19. De Bonis L, Koufos GD. 1991 The late Miocene small carnivores of the lower Axios valley (Macedonia-Greece). *Geobios* 24:361-379. DOI: 10.1016/S0016-6995(09)90015-8

20. De Bonis L, Koufos GD. 1994. Some Hyaenidae from the Late Miocene of Macedonia (Greece) and a contribution to the phylogeny of the hunting hyaenas. *Münchner Geowissenschaften Abhlungen* 26:81-96.

21. De Bonis L, Peigné S, Guya F, Taisso H, Likius A, Vignaud P, Brunet M. 2010 Hyaenidae (Carnivora) from the late Miocene of Toros-Menalla, Chad. *Journal of African Earth Sciences* 58:561-579. DOI: 10.1016/j.jafrearsci.2010.06.003

22. De Bonis L, Peigné S, Likius A, Makaye HT, Brunet M, Vignaud P. 2007. First occurrence of the ‘hunting hyena’Chasmaporthetes in the Late Miocene fossil bearing localities of Toros Menalla, Chad (Africa). *Bulletin de la Société géologique de France* 178:317-326. DOI: 10.2113/gssgfbull.178.4.317

23. De Bonis L. 2004. Carnivores hyaenidés du Miocène supérieur de Turquie. *Zona arqueológica* 4:108-117.

24. De Bonis L. 2005. Carnivora (Mammalia) from the late Miocene of Akkasdagı, Turkey. In: Sen S, ed. Geology, mammals and environments at Akkasdagı, late Miocene of Central Anatolia. *Geodiversitas* 27:567-590.

25. Del Campana D. 1914. La Lycyaena lunensis n. sp. dell'ossario pliocenico di Olivola (Val di Magra). *Paleonlographia Italica* 20:87-104.

26. Dubar M, Guerin C, Heintz E. 1978. Les nouveaux gisements villafranchiens du ravin de Cornillet (Moustiers Sainte-Marie, Alpes de Haute Provence, France) et leur contexte géologique. *Geobios* 11:367-381. DOI: 10.1016/S0016-6995(78)80036-9

27. Eisenmann V. 1988. Contributions a l'etude du gisement Miocene Superieur de Montredon (Herault). Les Grans Mammiferes. 2- Les Carnivores. *Palaeovertebrata* Mémorie extraordinaire 1988:15-42.

28. Ewer RF. 1954. The fossil carnivores of the Transvaal caves. The Hyaenidae of Kromdraai. *Proceedings of the Zoological Society of London. Blackwell Publishing Ltd* 124:565-585. DOI: doi.org/10.1111/j.1469-7998.1954.tb07798.x

29. Ewer RF. 1955. The fossil carnivores of the Transvaal caves. The Hyaenidae, other than Lycyaena, of Swartkrans and Sterkfontein. *Journal of Zoology* 124:815-837. DOI: 10.1111/j.1469-7998.1955.tb07819.x

30. Ewer RF. 1955. The fossil carnivores of the Transvaal caves. The lycyaenas of Sterkfontein and Swartkrans, together with some general considerations of the Transvaal fossil hyaenids. *Proceedings of the Zoological Society of London. Blackwell Publishing Ltd.* 124:839-857. DOI: 10.1111/j.1469-7998.1955.tb07820.x

31. Forsyth-Mayor CI. 1903. New Carnivora from the middle Miocene of La Grive St-Alban. *Geological Magazine, ns* 1:534-538.

32. Fourvel JB. 2012 Hyénidés modernes et fossiles d'Europe et d'Afrique: taphonomie comparée de leurs assemblages osseux. Thesis, Université Toulouse le Mirail-Toulouse II.

33. Gaillard C. 1899. Mammifères miocènes nouveaux ou peu connus de La Grive St-alban. *Archives Muséum Lyon* 7:1-79.

34. Galiano H, Frailey D. 1977. Chasmaporthetes kani, new species from China: with remarks on phylogenetic relationships of genera within the Hyaenidae (Mammalia, Carnivora). *American Museum novitates* 2632

35. Gaudry A. 1862 *Animaux fossiles et géologie de l'Attique*. Savy

36. Geraads D, Alemseged Z, Bobe R, Reed D. 2015. Pliocene Carnivora (Mammalia) from the Hadar Formation at Dikika, Lower Awash Valley, Ethiopia. *Journal of African Earth Sciences* 107:28-35. DOI: 10.1016/j.jafrearsci.2015.03.020

37. Geraads D. 1997. Carnivores du Pliocène terminalde Ahl al Oughlam (Casablanca, Maroc). *Geobios* 30:127-164. DOI: 10.1016/S0016-6995(97)80263-X

38. Haile-Selassie Y, Vrba ES, Bibi F. 2009. Carnivora. In: Woldegabriel G, ed. *Ardipithecus kadabba: late miocene evidence from the Middle Awash, Ethiopia*. Univ of California Press, 237-275. DOI: 10.1525/california/9780520254404.001.0001

39. Harris JM, Leakey MG, Brown FH. 1988. Stratigraphy and paleontology of Pliocene and Pleistocene localities west of Lake Turkana, Kenya. *Natural History Museum of Los Angeles County* 399:1-128.

40. Hernández-Pacheco E. 1930. Las grandes fieras de los yacimientos paleontológicos de Concud (Teruel). *Boletín de la Real Sociedad Española de Historia Natural* 30:149-158.

41. Hendey QB. 1973. Carnivore remains from the Kromdraai australopithecine site (Mammalia, Carnivora). *Annals of the Transvaal Museum* 8:99-112.

42. Howell FC, García N. 2007 Carnivora (Mammalia) from Lemudong’o (Late Miocene: Narok District, Kenya). *Kirtlandia* 56:121-139.

43. Howell FC, Petter G. 1980. The Pachycrocuta and Hyaena lineages (Plio-Pleistocene and extant species of the Hyaenidae). Their relationships with Miocene ictitheres: Palhyaena and Hyaenictitherium. *Geobios* 13:579-623. DOI: 10.1016/S0016-6995(80)80004-0

44. Howell FC, Petter G. 1985. Comparative observations on some middle and upper miocenehyaenids, Genera: PercrocutaKretzoi, AllohyaenaKretzoi, AdcrocutaKretzoi (Mammalia, Carnivora, Hyaenidae). *Geobios* 18:419-491. DOI: 10.1016/S0016-6995(85)80001-2

45. Hunt JR RM, Solounias N. 1991. Evolution of the aeluroid Carnivora. Hyaenid affinities of the Miocene carnivoran Tungurictis spocki from Inner Mongolia. *American Museum novitates* 3030

46. Kaya T, Geraads D, Tuna V. 2003. A new middle Miocene mammalian fauna from Mordoğan (Western Turkey). *Paläontologische Zeitschrift* 77:293-302. DOI: 10.1007/BF03006943

47. Kaya T, Geraads D, Tuna V. 2005. A new Late Miocene mammalian fauna in the Karaburun Peninsula (W Turkey). *Neues Jahrbuch für Geologie und Paläontologie-Abhandlungen* 236:321-349.

48. Kostopoulos DS, Sen S. 1999. Late Pliocene (Villafranchian) mammals from Sarikol Tepe, Ankara, Turkey. *Mitteilungen der bayerischen Staatssammlung für Paläontologie und historische Geologie* 39:165-202.

49. Koufos GD. 1992. The Pleistocene carnivores of the Mygdonia Basin (Macedonia, Greece). *Annales de Paléontologie* 78:205-257.

50. Koufos GD, Kostopoulos, D. 1997. New Carnivore material from the Plio-Pleistocene of Macedonia (Greece) with a description of a new canid. *Münchner Geowissenschaften Abhlungen* 34:33-63.

51. Koufos GD. 1993. Late Pliocene carnivores from western Macedonia (Greece). *Paläontologische Zeitschrift* 67:357-376. DOI: 10.1007/BF02990288

52. Koufos GD. 2000. Revision of the late Miocene carnivores from the lower Axios valley. *Münchener Geowissenschaften Abhanlungen* 39:51-92.

53. Koufos GD. 2006. The late Miocene vertebrate locality of Perivolaki, Thessaly, Greece. 4. Carnivora. *Palaeontographica Abteilung* 276:39-74.

54. Koufos GD. 2008. Carnivores from the early/middle Miocene locality of Antonios (Chalkidiki, Macedonia, Greece). *Geobios* 41:365-380. DOI: 10.1016/j.geobios.2007.05.003

55. Koufos GD. 2009. The Late Miocene Mammal Faunas of the Mytilinii Basin, Samos Island, Greece: New Collection. 5. Carnivora. *Beiträge zur Paläontologie* 31:57-105.

56. Koufos GD. 2011. The Miocene carnivore assemblage of Greece. *Estudios Geológicos* 67:291-320. DOI: 10.3989/egeol.40560.190

57. Koufos GD. 2012. A new protictithere from the late Miocene hominoid locality Ravin de la Pluie of Axios Valley (Macedonia, Greece). *Paläontologische Zeitschrift* 86:219-229. DOI: 10.1007/s12542-011-0126-9

58. Koufos GD. 2012. New material of Carnivora (Mammalia) from the Late Miocene of Axios Valley, Macedonia, Greece. *Comptes Rendus Palevol* 11:49-64. DOI: 10.1016/j.crpv.2011.09.004

59. Kovachev D. 2012. A complete skeleton of Adcrocuta eximia (Roth and Wagner, 1854) from the Upper Maeotian (Turolian) of Hadzhidimovo, SW Bulgaria. *Geologia Balkanica* 41:77-95.

60. Kuhn BF, Werdelin L, Hartstone-Rose A, Lacruz RS, Berger LR. 2011. Carnivoran remains from the Malapa hominin site, South Africa. *PLOS one* 6:e26940 DOI: 10.1371/journal.pone.0026940

61. Kuhn BF, Werdelin L, Steininger C. 2017. Fossil Hyaenidae from Cooper’s Cave, South Africa, and the palaeoenvironmental implications. *Palaeobiodiversity and Palaeoenvironments* 97:355-365. DOI: 10.1007/s1254

62. Kurtén B, Poulianos AN. 1981. Fossil Carnivora in Petralona Cave. Status of 1980 in The Third European Congress of Anthropology. Anthropos. *Etesio Organo tes Anthropologikes Etaireias Ellados Athinai* 8:9-56.

63. Kurtén B, Werdelin L. 1988. A review of the genus Chasmaporthetes Hay, 1921 (Carnivora, Hyaenidae). *Journal of Vertebrate Paleontology* 8:46-66. DOI: 10.1080/02724634.1988.10011683

64. Kurtén B. 1956. The status and affinities of Hyaena sinensis Owen and Hyaena ultima Matsumoto. *American Museum novitates* 1764

65. Liu J, Zhao L, Chen J, Wang X, Cai H, Zhang Z. 2011. Age and environment of the giant fauna of Bijie, Qujie, Guizhou. *Quaternary study* 31:654-666. DOI: 10.3969/j.issn.1001-7410.2011.04.

66. Luptak P. 1995. Ictitherium viverrinum (Carnivora, Hyaenidae) from Upper Miocene of Western Slovakia. *Geologica Carpathica-Bratislava* 46:349-356.

67. Maroto J, Galobart A, Antón M. 2003. Hiénidos y cánidos de los yacimientos de Incarcal (Girona, NE de la Península Ibérica): una aproximación a la paleobiología del hiénido del Pleistoceno inferior. *Paleontologia i Evolució* 34:79-98.

68. Mayda S, Koufos GD, Kaya T, Gul A. 2015. New carnivore material from the Middle Miocene of Turkey. Implications on biochronology and palaeoecology. *Geobios* 48:9-23. DOI: 10.1016/j.geobios.2014.11.001

69. Mol D, de Vos J. 1995. De hyena uit de Oosterschelde. *Grondboor en Hamer* 49:139-149.

70. Montoya P. 1997. Los hiénidos (Carnivora, Mammalia) del Mioceno Superior (Turoliense inferior) de Crevillente 2 (provincia de Alicante, España). *Revista Española de Paleontología* 12:265-273.

71. Morales J, Senut B, Pickford M. 2011. Crocuta dietrichi from Meob, Namibia: implications for the age of the Tsondab Sandstone in the coastal part of Namibia. *Estudios Geológicos* 67:207-215.

72. Moyà JP. 1982. HYAENIDAE (Carnivora, Mammalia) del Pleistoceno inferior de la Cueva Victoria. *Endins: publicació d'espeleologia* 9:45-48.

73. Mutter RJ, Berger LR, Schmid P. 2001. New evidence of the giant hyaena, Pachycrocuta brevirostris (Carnivora, Hyaenidae), from the Gladysvale Cave Deposit (Plio-Pleistocene, John Nash Nature Reserve, Gauteng, South Africa). *Palaeontologia Africana* 37:103-113.

74. O'regan HJ, Menter CG. 2009. Carnivora from the Plio-Pleistocene hominin site of Drimolen, Gauteng, South Africa. *Geobios* 42:329-350. DOI: 10.1016/j.geobios.2009.03.001

75. Özkurt SÖ, Güleç E, Erkman AC. 2015. Carnivores from the Late Miocene locality of Hayranlı (Hayranlı, Sivas, Turkey). *Turkish Journal of Zoology* 39:842-867. DOI: 10.3906/zoo-1407-38

76. Peigné S, Salesa MJ, Antón M, Morales J. 2006. New data on carnivores from the Middle Miocene (Upper Aragonian, MN 6) of Arroyo del Val area (Villafeliche, Zaragoza Province, Spain). *Estudios Geológicos* 62:359-374. DOI: 10.3989/egeol.0662131

77. Petricci M, Cipullo A, Martínez-Navarro B, Rook L, Sardella R. 2013. The late Villafranchian (Early Pleistocene) carnivores (Carnivora, Mammalia) from Pirro Nord (Italy). *Palaeontographica Abteilung* 2013:113-145. DOI: 10.1127/pala/298/2013/113

78. Pickford M, Soria D. 2005. Carnivores from the late Miocene and basal Pliocene of the Tugen Hills, Kenya. *Revista de la Sociedad Geológica de España* 18:39-61.

79. Qiu Z, Ye J, Cao J. 1988. A new species of Percrocuta from Tongxin, Ningxia. *Vertebrata PalAsiatica* 26:116-127.

80. Randall RM. 1981. Fossil hyaenidae from the Makapansgat limeworks deposit, South Africa. *Palaeontologia africana* 24:75-85.

81. Rook L, Ferretti MP, Arca M, Tuveri C. 2004. Chasmaporthetes melei N. sp, an endemic hyaenid (Carnivora, Mammalia) from the Monte Tuttavista fissure fillings (Late Pliocene to Early Pleistocene; Sardinia, Italy). *Rivista italiana di Paleontologia e Stratigrafia* 110:707-714. DOI: 10.13130/2039-4942/5833

82. Roussiakis SJ, Theodorou GE. 2003. Carnivora from the late Miocene of Kerassia (Northern Euboea, Greece). *Deinsea* 10:469-497.

83. Semenov YA. 1989. *Ictitheres and morphologically related hyaenas from the Neogene of the USSR*. Naukova Dumka, Kiev.

84. Şenyürek M. 1985. A study of the remains of Crocuta from the Küçükyozgat district. *D. Rev. de la Fac. Long. Hist. et Geog., Univ. Ankara* 12:29-74. DOI: 10.1501/Dtcfder_0000001083

85. Soria D. 1980. " Percrocuta y Adcrocuta"(Hyaenidae, Mammalia) en el Mioceno superior del área de Teruel. *Estudios geológicos* 36:143-162.

86. Sotnikova MV, Titov V. 2009. Carnivora of the Tamanian faunal unit (the Azov Sea area). *Quaternary International* 201:43-52. DOI: 10.1016/j.quaint.2008.05.019

87. Sotnikova MV, Baigusheva VS, Titov VV. 2002. Carnivores of the Khapry faunal assemblage and their stratigraphic implications. *Stratigraphy and Geological Correlation* 10:375-390.

88. Sotnikova MV. 1994. The genus Chasmaporthetes Hay, 1921 from the Pliocene of Russia, Ukraine, Mongolia and Tadzhikistan. In: Tatarinov LP, ed. *Palaeotheriology. Nauka, Moscow*, 113-139.

89. Suraprasit K, Jaeger JJ, Chaimanee Y, Benammi M, Chavasseau O, Yamee C, Tian P, Panha S. 2015. A complete skull of Crocuta crocuta ultima indicates a late Middle Pleistocene age for the Khok Sung (northeastern Thailand) vertebrate fauna. *Quaternary International* 374:34-45. DOI: 10.1016/j.quaint.2014.12.062

90. Torregrosa V. 2008. Análisis ecomorfológicos de grandes carnívoros del plio-pleistoceno (familias canidae, hyanidae y felidae). D. Eco. y Geo. Thesis, Universidad de Málaga.

91. Tseng ZJ, Chang CH. 2007. A study of new material of Crocuta crocuta ultima (Carnivora: Hyaenidae) from the Quaternary of Taiwan. *Collection and Research*, 20:9-19.

92. Tseng ZJ, Jin CZ, Liu JY, Zheng LT, Sun CK. 2008. Fossil Hyaenidae (Mammalia: Carnivora) from Huainan, Anhui Province, China. *Ancient Vertebrate Journal* 46:133-146.

93. Tseng ZJ, Li Q, Wang X. 2013. A new cursorial hyena from Tibet, and analysis of biostratigraphy, paleozoogeography, and dental morphology of Chasmaporthetes (Mammalia, Carnivora). *Journal of Vertebrate Paleontology* 33:1457-1471. DOI: 10.1080/02724634.2013.775142

94. Tseng ZJ, Wang X. 2007. The first record of the late Miocene Hyaenictitherium hyaenoides Zdansky (Carnivora: Hyaenidae) in Inner Mongolia and an evaluation of the genus. *Journal of Vertebrate Paleontology* 27:699-708. DOI: 10.1671/0272-4634(2007)27[699:TFROTL]2.0.CO;2

95. Turner A, Antón M. 1996. The giant hyaena, Pachycrocuta brevirostris (Mammalia, Carnivora, Hyaenidae). *Geobios* 29:455-468. DOI: 10.1016/S0016-6995(96)80005-2

96. Turner A. 1986. Miscellaneous carnivore remains from Plio-Pleistocene deposits in the Sterkfontein valley (Mammalia: Carnivora). *Annals of the Transvaal Museum* 34:203-226.

97. Turner A. 1987. New fossil carnivore remains from the Sterkfontein hominid site (Mammalia: Carnivora). *Annals of the Transvaal Museum* 34:319-347.

98. Turner A. 1997. Further remains of Carnivora (Mammalia) from the Sterkfontein hominid site. *Palaeontologia Africana* 34:115-126.

99. Villalta-Comella JF. Contribución al conocimiento de la fauna de mamíferos fósiles del Plioceno de Villaroya (Logroño). Thesis. Boletín del Instituto Geológico y Minero de España vol. 64:1-203

100. Vinuesa V, Madurell-Malapeira J, Werdelin L, Robles JM, Obradó P, Alba DM. 2017. A New Skull of Hyaenictis Gaudry, 1861 (Carnivora, Hyaenidae) Shows Incipient Adaptations to Durophagy. *Journal of mammalian evolution* 24:207-219. DOI: 10.1007/s1091

101. Viranta S, Werdelin L. 2003. Carnivora from the Sinap Formation, Turkey. In: Fortelius M, Kappelman J, Bernor RL, Sen S, ed. *The Miocene Sinap Formation, Turkey*. New York: Columbia University Press 178-193.

102. Wang X, Ye J, Meng J, Wu WY, Liu LP, Bi SD. 1998. Carnivora from middle Miocene of northern Junggar Basin, Xinjiang Autonomous Region, China. *Vertebrata PalAsiatica* 36:218-243.

103. Wang X. 2004. New materials of Tungurictis (Hyaenidae, carnivora) from tunggur formation, Nei mongol. *Vertebrata PalAsiatica* 42:144-153.

104. Werdelin L, Dehghani R. 2011. Carnivora. In: *Paleontology and geology of Laetoli: Human evolution in context*. Springer Netherlands, 189-232.

105. Werdelin L, Kurtén B. 1999. Allohyaena (Mammalia: carnivora): giant hyaenid from the Late Miocene of Hungary. *Zoological Journal of the Linnean Society* 126:319-334. DOI: 10.1111/j.1096-3642.1999.tb01374.x

106. Werdelin L, Lewis ME. 2008. New species of Crocuta from the early Pliocene of Kenya, with an overview of early Pliocene hyenas of eastern Africa. *Journal of Vertebrate Paleontology* 28:1162-1170. DOI: 10.1671/0272-4634-28.4.1162

107. Werdelin L, Turner A, Solounias N. 1994. Studies of fossil hyaenids: the genera Hyaenictis Gaudry and Chasmaporthetes Hay, with a reconsideration of the Hyaenidae of Langebaanweg, South Africa. *Zoological Journal of the Linnean Society* 111:197-217. DOI: 10.1111/j.1096-3642.1994.tb01483.x

108. Werdelin L. 1988. Studies of fossil hyaenas: the genera Thalassictis Gervais ex Nordmann, Palhyaena Gervais, Hyaenictitherium Kretzoi, Lycyaena Hensel and Palinhyaena Qiu, Huang & Guo. *Zoological Journal of the Linnean Society* 92:211-265. DOI: 10.1111/j.1096-3642.1988.tb01512.x

109. Werdelin L. 1988. Studies of fossil hyaenids: the genera Ictitherium Roth & Wagner and Sinictitherium Kretzoi and a new species of Ictitherium. *Zoological journal of the Linnean Society* 93:93-105.

110. Werdelin L. 1999. Pachycrocuta (hyaenids) from the Pliocene of east Africa. *Paläontologische Zeitschrift* 73:157-165. DOI: 10.1007/BF02987989

111. Werdelin L. 1999. Studies of fossil hyaenas: affinities of Lycyaenops rhomboideae Kretzoi from Pestlörinc, Hungary. *Zoological Journal of the Linnean Society* 126:307-317. DOI: 10.1111/j.1096-3642.1999.tb01373.x

112. Werdelin L. 2003. Carnivores from the Kanapoi hominid site, Turkana Basin, northern Kenya. *Contributions in Science* 498:115-132.

113. Werdelin L. 2003. Mio-Pliocene Carnivora from Lothagam, Kenya. In Leakey MG, Harris JM, ed. *Lothagam: the dawn of humanity in eastern Africa*. Columbia University Press 261-328. DOI: 10.7312/leak11870

114. Werdelin L. 2005. Carnivora from the late Miocene of Lantian, China. *Ancient Vertebrate Journal* 43:256-2717.

115. Zapfe H. 1948. Neue Funde von Raubtieren aus dem Unterpliozän des Wiener Beckens. *Sitzingsberichte der Österreichischen Akademie der Wissendchaften, mathematisch-naturwissenschaftliche Klasse* 157:243-262.

116. Zhang ZQ. 2001. Fossil mammals of Early Pleistocene from Ningyang, Shandong Province. *Vertebrata PalasiAtica* 39:140-154.

117. Zhan-Xiang Q, Zhi-Hui G, Wei-long H. 1979. Hyaenidae of the Qingyang (K’ingyang) Hipparion Fauna. *Vertebrata PalAsiatica* 3:200-227.

118. Zheng SH, 1982. Some Pliocene mammalian fossils from Songshan-2 and-3 (Tianzhu, Gansu) and the Songshan fauna. *Vertebrata PalAsiatica* 20:216-227.

119. Zhu M, Yaling Y, Yihong, L, Zhilu T, Dagong Q, Changzhu J. 2015. The new Carnivore remains from the Early Pleistocene Yanliang Gigantopithecus fauna, Guangxi, South China. *Quaternary International* 434:17-24. DOI: 10.1016/j.quaint.2015.01.009

**MSNF (Museo di Storia Naturale di Firenze)**
