# Supplementary material for: Characterizing ecomorphological patterns in hyenids: a multivariate approach using postcanine dentition
Source: PeerJ. 2019 Jan 11;6:e6238. doi: 10.7717/peerj.6238 (PMC6330948; doi:10.7717/peerj.6238)
Supplement: Supplemental Information 2 [file peerj-07-6238-s002.docx]

***Pachycrocuta brevirostris***

IGF 12470, IGF 12478, IGF 835, IGF 12738, IGF 12739, IGF 840, IGF 1212v, IGF 841, IGF 836, IGF 1206v, IGF 849, IGF 837, IGF 1210v, IGF 1211v, IGF 1209v, IGF 842, IGF 1213v, IGF 1206v, IGF 845, IGF 843, IGF 834, IGF 838, IGF 10762, IGF 494v, IGF 710v, IGF 4384, IGF 699v, IGF 839

***Pliocrocuta perrieri***

IGF 10763, IGF 12471, IGF 4853v, IGF 4854v, IGF 5504v, IGF 5765v, IGF 1391vA, IGF 1391vB, IGF 1394v

***Chasmaporthetes lunensis***

IGF 4377, IGF 17222

***Adcrocuta eximia***

IGF 13582

***Crocuta crocuta* (fossil)**

IGF 6593v, IGF 6592v, IGF 6591v, IGF 6562v, IGF 6572v, IGF 6571v, IGF 4860v, IGF 4725, IGF 6702v, IGF 14714, IGF 14715, IGF 4724, IGF 102799, IGF 7398v, IGF 102800, IGF 12483
